# Synthetic data in rheumatology: a systematic literature review

**DOI:** 10.1093/rap/rkag114

**Published:** 2026-09-25

**Authors:** Vincenzo Venerito, Maria Morrone, Sergio Del Vescovo, Emre Bilgin, Latika Gupta, Giuseppe Lopalco, Florenzo Iannone

**Affiliations:** Rheumatology Unit, Department of Precision and Regenerative Medicine–Ionian Area (DiMePRe-J), University of Bari “Aldo Moro”, Bari, Italy; Rheumatology Unit, Department of Precision and Regenerative Medicine–Ionian Area (DiMePRe-J), University of Bari “Aldo Moro”, Bari, Italy; Rheumatology Unit, Department of Precision and Regenerative Medicine–Ionian Area (DiMePRe-J), University of Bari “Aldo Moro”, Bari, Italy; Division of Rheumatology, Department of Internal Medicine, Faculty of Medicine, Sakarya University, Sakarya, Turkey; Department of Rheumatology, Royal Wolverhampton NHS Trust, Wolverhampton, UK; Institute of Inflammation and Ageing, University of Birmingham, Birmingham, UK; Francis Crick Institute, Camden, London, UK; Rheumatology Unit, Department of Precision and Regenerative Medicine–Ionian Area (DiMePRe-J), University of Bari “Aldo Moro”, Bari, Italy; Rheumatology Unit, Department of Precision and Regenerative Medicine–Ionian Area (DiMePRe-J), University of Bari “Aldo Moro”, Bari, Italy

**Keywords:** synthetic data, rheumatology, systematic review, generative adversarial networks, digital twins, privacy-preserving data, healthcare, data augmentation, large language models, diffusion models

## Abstract

**Objective:**

To systematically review the methods, validation frameworks, applications and regulatory landscape of synthetic data generation in healthcare, with a specific focus on rheumatology.

**Methods:**

We searched PubMed/MEDLINE, Embase and arXiv (January 2016–January 2026). From 1099 database records and 7 expert-identified studies, 701 remained after removing 405 duplicates. Title and abstract screening excluded 353 records; full-text assessment of 348 articles excluded 120 (E1: *n* = 62; E2: *n* = 1; E3: *n* = 23; E4: *n* = 34), yielding 228 studies. Quality assessment of 13 rheumatology-specific studies using a 10-item checklist revealed 1 high-quality, 8 moderate-quality, and 4 low-quality studies, with systematic gaps in privacy evaluation (2/13) and reproducibility (1/13).

**Results:**

The 228 included studies encompassed six generation-method categories (generative adversarial networks, diffusion models, large language models, statistical methods, rule-based simulators and hybrid approaches) and five thematic categories (digital twins, privacy-preserving methods, reviews, validation frameworks and healthcare applications), detailed in the supplementary methods. Validation remained inconsistent, with only a minority evaluating fidelity, utility and privacy simultaneously. In rheumatology, 13 studies spanned rheumatoid arthritis (*n* = 3), osteoarthritis (*n* = 3), Sjogren’s syndrome (*n* = 2), systemic sclerosis (*n* = 2), systemic lupus erythematosus (*n* = 2) and ankylosing spondylitis (*n* = 1). Only 2 of 13 formally evaluated privacy and only 1 shared code for reproducibility.

**Conclusion:**

Synthetic data hold substantial promise for rheumatology, particularly for augmenting small cohorts in low-prevalence diseases, enabling privacy-preserving registry sharing and simulating clinical trial populations. Realizing this potential requires standardized validation, greater attention to privacy and reproducibility and regulatory clarity on synthetic health data.

Key messages

- Synthetic data are a promising but methodologically immature enabling technology for rheumatology artificial intelligence.
- Rheumatology use remains sparse, with only 13 studies showing systematic gaps in privacy and reproducibility.
- Realising this potential requires standardised validation and regulatory clarity on synthetic health data.

## Introduction

Synthetic data are artificially generated information that reproduce the statistical patterns found in real patient records without corresponding to any actual individual [1]. Clinical research in rheumatology is increasingly constrained by two opposing forces: the growing demand for large, representative datasets to train artificial intelligence (AI) models and inform precision medicine and the tightening of privacy regulations that restrict how patient data can be collected, stored and shared. Synthetic data have emerged as a potential solution to this tension.

A key distinction exists between fully synthetic data, where every record is generated de novo, and partially synthetic data, where only selected sensitive attributes are replaced with artificial values while the remaining clinical information is retained [2, 3]. Fully synthetic datasets offer stronger privacy protection but may sacrifice analytical precision, whereas partially synthetic datasets preserve more of the original data structure but carry residual re-identification risk. Synthetic data generation should also be distinguished from data augmentation: augmentation expands an existing dataset by transforming or resampling real records, e.g. through oversampling techniques such as the synthetic minority oversampling technique (SMOTE) or geometric transformations, whereas synthetic data generation creates entirely new records from a learned model of the underlying data distribution [4].

The importance of synthetic data in healthcare stems from several converging challenges. Privacy regulations such as the European Union (EU) General Data Protection Regulation (GDPR) and the US Health Insurance Portability and Accountability Act (HIPAA) impose significant restrictions on sharing patient-level data across institutions and national borders. Many clinical datasets are too small to support modern machine learning approaches, and real-world clinical data frequently suffers from class imbalance, where clinically important events such as macrophage activation syndrome, scleroderma renal crisis or pulmonary arterial hypertension in SSc are represented by only a handful of cases, with event rates often <5% even in dedicated registries. Synthetic data offer the potential to address all three challenges simultaneously: enabling broader data sharing without exposing individual patients, augmenting small datasets to meet the requirements of modern analytical methods and oversampling underrepresented clinical scenarios.

The methods used to generate synthetic data have evolved from straightforward statistical techniques to sophisticated deep learning approaches [4]. These include Bayesian networks and copula models [5, 6]; generative adversarial networks (GANs), which use two competing neural networks in a competitive training process that produces increasingly realistic synthetic data, with variants such as conditional tabular GAN designed for mixed clinical data types; diffusion models that reverse a gradual noising process, producing higher-quality outputs than GANs while avoiding mode collapse, though at greater computational cost; large language models (LLMs) that generate clinical narratives but carry hallucination risk [7, 8]; digital twin frameworks that simulate disease biology rather than learning from data [9–11] and hybrid approaches combining multiple paradigms [12, 13].

Rheumatology presents a compelling case for synthetic data adoption, as the field’s defining characteristics align closely with the problems these methods are designed to solve. The field encompasses >200 autoimmune and musculoskeletal diseases, many of which are low-prevalence, meaning that even large academic centres may accumulate insufficient cases for machine learning. Multinational registries such as the EULAR Scleroderma Trials and Research group (EUSTAR) and Spanish Registry for Adverse Events of Biological Therapy in Rheumatic Diseases (BIOBADASER) pool data across institutions, but sharing patient-level data across borders remains legally complex. Phenotypic heterogeneity further compounds the challenge: SLE, for instance, can present with diverse organ involvement, creating a vast multidimensional clinical space that no single centre can adequately sample. Overlap syndromes further blur diagnostic boundaries, meaning that datasets must be not only large but also clinically diverse to support generalizable research. The field increasingly relies on multimodal data, clinical scores, laboratory biomarkers, imaging and genomic profiles, while the longitudinal nature of rheumatic diseases introduces additional complexity for data collection and sharing. Additionally, clinical trials face persistent recruitment difficulties for rare conditions, where synthetic control arms could supplement real data.

This review systematically examines synthetic data generation in healthcare with a specific focus on rheumatology. To our knowledge, no previous systematic review has examined this intersection. We organize findings by generation methods, validation frameworks, healthcare and rheumatology-specific applications, privacy and regulatory considerations and intrinsic methodological limitations.

## Methods

We conducted a systematic search of PubMed/MEDLINE, Embase and arXiv for studies published between January 2016 and January 2026. The search strategy combined terms for synthetic data generation with healthcare and rheumatology-specific terms; full search strings are provided in Supplementary Material S1. Four complementary search streams were executed: stream A targeted PubMed with rheumatology-specific terms (*n* = 144), stream B searched PubMed broadly for synthetic data in healthcare (*n* = 314), stream C queried arXiv through four parallel searches (*n* = 250) and stream D adapted the PubMed strategy to Embase (*n* = 391). Additionally, seven domain-expert-identified studies were included. Key EU regulations [GDPR, AI Act, European Health Data Space (EHDS), Clinical Trials Regulation, Medical Device Regulation] are cited as primary legal sources in Section 7. This review was registered on INPLASY (International Platform of Registered Systematic Review and Meta-Analysis Protocols; registration number: INPLASY202630024; DOI: 10.37766/inplasy2026.3.0024).

Studies were eligible if they focused on synthetic data generation, evaluation, privacy-preserving data sharing, digital twins or regulatory frameworks in a healthcare context and were available as full papers. Studies were excluded if they merely used data augmentation as preprocessing or applied only standard geometric augmentation (E1), provided only an abstract or poster (E2), constituted commentary without original data (E3) or were not about healthcare or clinical data, including studies addressing only drug or molecular design (E4).

Records were managed in Zotero (https://www.zotero.org/) and screened independently by two reviewers in two stages, with disagreements resolved through discussion. Title and abstract screening excluded 353 records. Full-text assessment of 348 articles excluded 120 (E1: *n* = 62, E2: *n* = 1, E3: *n* = 23, E4: *n* = 34), yielding 228 studies for qualitative synthesis. For each included study, one reviewer extracted and a second reviewer verified the study design, generation method, data type, application domain, validation metrics, privacy evaluation, key findings and limitations. Each study was then assigned to one of 11 categories by primary focus: 6 generation method categories (GANs, which pit two competing neural networks against each other to produce realistic tabular or imaging data; diffusion models, which iteratively denoise to reach high fidelity while avoiding mode collapse; LLMs, which generate clinical text and records but carry hallucination risk; statistical methods, such as Bayesian networks, copulas and resampling; rule-based simulators, which are mechanistic, knowledge-driven models that generate data from disease rules rather than learning from data; and hybrid approaches, which combine two or more of these paradigms) and 5 thematic categories (digital twins and virtual patients, privacy-preserving methods, reviews and surveys, validation frameworks, healthcare applications), as detailed in Supplementary Table S1.

A systematic evaluation of validated risk of bias tools (RoB 2 [14], ROBINS-I [15], QUADAS-2 [16], PROBAST [17], PROBAST+AI [18], QUADAS-AI [19]) determined that none are applicable to synthetic data generation studies. These instruments were built to appraise risk of bias in studies of human participants with defined clinical outcomes: ROBINS-I [15] targets non-randomised intervention studies through domains such as confounding, participant selection and deviation from the intended intervention, whereas PROBAST [17] targets clinical prediction-model studies through the domains of participants, predictors, outcome and analysis. A synthetic data generation study, by contrast, has no clinical intervention, no patient-level outcome and no prognostic prediction task, so the signalling questions that drive these tools do not map onto its design. For the 13 rheumatology-specific studies (Section 6), a purpose-designed 10-item checklist was developed, informed by PROBAST [17], TRIPOD+AI [14] and synthetic data evaluation criteria [20]. The items assessed study design and data description (Q1, Q2); methodological rigor (Q3); evaluation completeness covering fidelity, utility and privacy (Q4–Q6); comparison and clinical relevance (Q7, Q8) and limitations and reproducibility (Q9, Q10). This author-derived checklist has not undergone formal validation and was used as a structured descriptive assessment rather than as a validated risk of bias instrument. Two reviewers independently scored each item as yes, partial, or no, with disagreements resolved by consensus. Overall quality was rated as high (seven or more yes), moderate (four to six yes) or low (three or fewer yes). Full results are presented in Supplementary Table S1. No formal quality assessment was performed for the remaining 215 studies, as no validated instrument exists for computational methodology studies; this limitation is acknowledged in Section 9.

### Ethics approval

This study is a systematic review based on previously published literature and did not involve human participants or identifiable data, therefore institutional review board approval was not required.

## Results

### Study selection

From 1099 database records (PubMed/MEDLINE: *n* = 458, Embase: *n* = 391, arXiv: *n* = 250) and 7 expert-identified studies, 405 duplicates were removed, leaving 701 records for title and abstract screening. Of these, 353 were excluded and 348 reports were sought for full-text retrieval (all retrieved). Full-text assessment excluded 120 studies (data augmentation as preprocessing only: *n* = 62, conference abstract only: *n* = 1, commentary/editorial without original data: *n* = 23, not about healthcare/clinical data: *n* = 34), yielding 228 studies included in the qualitative synthesis (Fig. 1).

**Figure 1:**
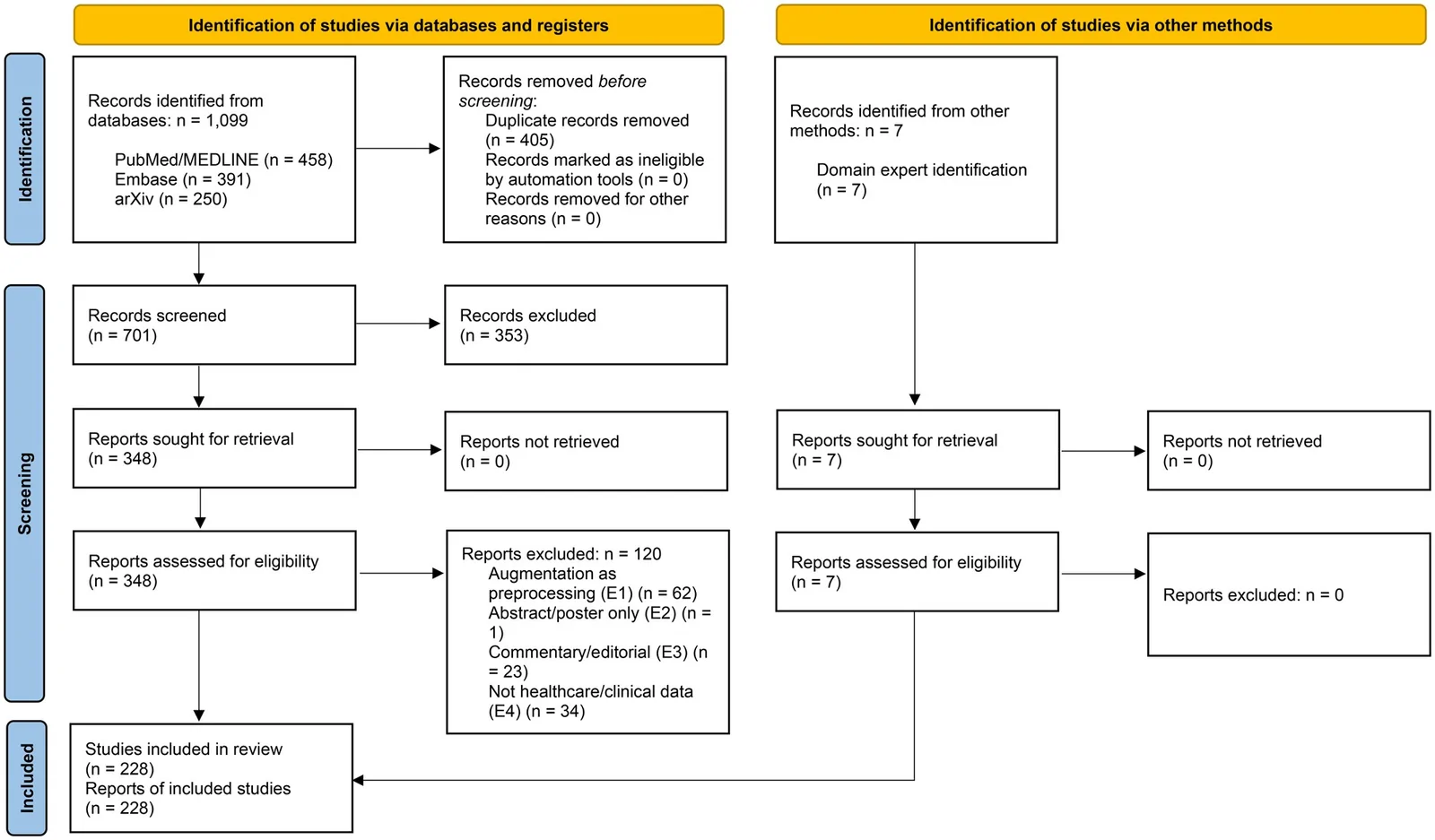
PRISMA 2020 flow diagram for the systematic review of synthetic data in rheumatology. From 1099 database records and 7 expert-identified studies, 228 studies were included in the qualitative synthesis after removing 405 duplicates, excluding 353 at title/abstract screening and excluding 120 at full-text assessment

Across the 228 included studies, six methodological categories were identified [GANs (*n* = 27), diffusion models (*n* = 8), LLMs (*n* = 23), statistical methods (*n* = 12), rule-based simulators (*n* = 11) and hybrid approaches (*n* = 16)] alongside five thematic categories [digital twins and virtual patients (*n* = 37), privacy-preserving methods (*n* = 32), reviews and surveys (*n* = 31), validation frameworks (*n* = 25) and healthcare applications (*n* = 6)]. A comprehensive description of generation methods, validation frameworks and healthcare applications with full citations is provided in the supplementary methods and Supplementary Table S2. Comprehensive evaluation across the fidelity–utility–privacy triad was the exception rather than the rule. Most studies reported only one or two of the three dimensions, typically utility (performance on downstream tasks) and, less often, fidelity (statistical similarity to real data), while privacy (resistance to re-identification) was the dimension least frequently and least rigorously assessed, even among studies generating patient-level data. Only a minority evaluated all three dimensions simultaneously. Because so few studies applied the full triad, and because those that did used heterogeneous metrics and thresholds, head-to-head comparison of generation methods across the corpus was substantially hampered.

### Applications and opportunities in rheumatology

Despite the rapid growth of synthetic data in healthcare generally, rheumatology-specific applications remain sparse but substantive. We identified 13 studies spanning six disease areas (Table 1). This underrepresentation, only 13 of 228 studies, reflects several converging factors. Most rheumatic diseases are individually low-prevalence, so single-centre datasets are small and fragmented, offering limited raw material for method development. Multinational registries such as EUSTAR and BIOBADASER do pool cases, but cross-border patient-level data sharing remains legally constrained, precisely one of the barriers synthetic data is meant to relieve. The field also has comparatively few dedicated computational and data-science groups and adopted machine learning later than radiology or oncology, from which most methodological studies in this review originate. The phenotypes rheumatologists work with are heterogeneous, longitudinal and multimodal and are therefore harder to model than the cross-sectional, single-modality data used in many benchmark applications. Finally, the very data scarcity that motivates synthetic data in rheumatology also limits the training data needed to build and validate good generators, creating a bottleneck that has slowed uptake in the field.

**Table 1:** Summary of 13 rheumatology-specific synthetic data studies.

Reference	Author (year)	Disease	Method	Application	Key finding
[11]	Zerrouk (2024)	RA	Digital twin (Boolean models)	Virtual synovial joint modelling	Modular multicellular virtual twin of RA synovial joint
[10]	Bedathuru (2024)	RA	Mechanistic multiscale model	Virtual patient populations for drug development	Generated virtual RA patients responding to MTX and TNFi
[9]	Paul (2024)	RA	SBI + QSP modelling	In silico clinical trials	Virtual RA patients with PK/PD integration
[21]	Prezja (2022)	OA	GAN	Synthetic knee X-ray generation	Synthetic images deceived experts in visual Turing test
[22]	Jayakumar (2025)	OA	Digital twin decision aid	RCT of shared decision-making	Improved decision quality and patient experience
[23]	Coleman (2025)	OA	Synthetic augmentation	Biomarker variability analysis	Identified distinct inflammatory phenotypes via clustering
[24]	Pezoulas (2023)	SS	Data augmentation	MALT lymphoma classification	Boosted classification performance in primary SS
[5]	Pezoulas (2021)	SS	BN + ANN + decision trees	Disease classification pipeline	Improved accuracy through multimethod augmentation
[25]	Jiang (2022)	SSc	Model-targeted masking + MI	Privacy-preserving registry sharing	Balanced inferential integrity with disclosure risk
[26]	Jia (2024)	SSc	Synthetic augmentation	Automated CT scoring of ILD	Improved scoring agreement with expert radiologists
[13]	Xie (2024)	SLE	VAE	Treatment outcome prediction	Predicted outcomes for SLE-associated ITP
[27]	Liu (2023)	SLE	GAN (TMM-Nets)	Retinopathy diagnosis	Multi- to monomodal generation from OCT and fundus
[28]	Sun (2023)	AS	ac-GAN	Privacy-preserving MRI sharing	Synthetic vertebral unit MRI with fidelity and privacy metrics

ac-GAN: auxiliary classifier GAN; ANN: artificial neural network; BN: Bayesian network; MI: multiple imputation; ILD: interstitial lung disease; ITP: immune thrombocytopenia; OCT: optical coherence tomography; PK/PD: pharmacokinetic/pharmacodynamic; QSP: quantitative systems pharmacology; RCT: randomized controlled trial; SBI: simulation-based inference; TNFi: tumour necrosis factor inhibitor; VAE: variational autoencoder.

RA leads the disease-specific literature with three studies representing some of the most sophisticated synthetic data work in the field. Zerrouk *et al*. [11] constructed a modular, multicellular virtual twin of the RA synovial joint using executable Boolean models. Bedathuru *et al*. [10] developed a mechanistic multiscale model generating virtual patient populations responding to methotrexate and TNF inhibitors for late-stage drug development. Paul *et al*. [9] combined simulation-based inference with quantitative systems pharmacology (QSP) modelling for in silico clinical trials integrating pharmacokinetic and pharmacodynamic parameters. Together, these illustrate a progression from molecular-level modelling through disease-scale simulation to clinical trial applications, suggesting that RA may serve as a prototype for digital twin development in other rheumatic diseases.

Three studies address OA, spanning imaging synthesis, clinical decision support and biomarker analysis. Prezja *et al*. [21] generated synthetic knee OA X-rays using GANs that deceived medical experts in a visual Turing test, demonstrating both image fidelity and augmentation potential for training diagnostic classifiers. Jayakumar *et al*. [22] conducted the only randomized clinical trial in this review, testing a digital twin-enabled decision aid for knee OA care and demonstrating improved shared decision-making outcomes and patient experience. Coleman *et al*. [23] applied synthetic data generation to analyse patient-specific variability in IL-6 and MPO responses, using clustering analysis to identify distinct inflammatory phenotypes. The OA studies are notable for their methodological diversity and direct clinical relevance, with the Jayakumar *et al*. [22] trial providing the only level-1 evidence for a synthetic data application in any rheumatic disease.

The remaining diseases are each represented by one or two studies. In SS, Pezoulas *et al*. [24] boosted mucosa-associated lymphoid tissue (MALT) lymphoma classification through augmentation, and in a separate study [5] developed a multimethod classification pipeline. In SSc, Jiang *et al*. [25] applied privacy-preserving methods to Canadian Scleroderma Research Group registry data, while Jia *et al*. [26] used augmentation for automated CT scoring of interstitial lung disease. Xie *et al*. [13] applied variational autoencoders for SLE-associated immune thrombocytopaenia prediction and Liu *et al*. [27] developed GAN-based multimodal retinopathy diagnosis. Sun *et al*. [28] generated synthetic MRIs for privacy-preserving sharing in AS.

Quality assessment using our 10-item checklist (Supplementary Table S1) revealed one high-quality study [10], eight moderate quality [9, 11, 21–23, 25, 26, 28] and four low quality [5, 13, 24, 27]. Study design and clinical relevance were generally well-addressed (Q1: 9/13 yes; Q8: 8/13 yes). However, three systematic weaknesses emerged. Privacy evaluation was infrequent, with only Jiang *et al*. [25] and Sun *et al*. [28] formally assessing re-identification risk, despite most studies generating patient-level data. Reproducibility was poor, with only 1 of 13 sharing code or data (Q10). Limitations were inadequately discussed (Q9: only 1/13 yes). Fidelity evaluation was mixed (Q4: 4/13 yes, 3/13 partial), while utility was more consistently addressed (Q5: 6/13 yes, 6/13 partial).

While these studies demonstrate technical feasibility, several domain-specific challenges remain. The phenotypic complexity of rheumatic diseases, where conditions like SLE feature subtypes and overlap syndromes, creates profiles exceeding the capacity of most generative models. The longitudinal nature of these diseases requires temporal methods [29–34] substantially more demanding than cross-sectional approaches. Multimodal data integration across clinical, imaging and genomic modalities remains largely unsolved [35, 36]. Low-prevalence manifestations within already low-prevalence diseases create severe class imbalance that augmentation could address, yet generating examples of uncommon events is itself harder precisely because training data are limited [37, 38]. Clinical plausibility must extend beyond statistical fidelity; domain expert validation, seldom performed [20, 39], is essential. The regulatory classification of synthetic health data remains unresolved [40–45].

Despite these challenges, substantial opportunities remain. Synthetic augmentation of registries like EUSTAR and BIOBADASER could expand training data. Virtual populations could simulate trial arms for low-prevalence diseases [9, 10]. GAN-augmented imaging could improve sacroiliitis detection and erosion scoring. Temporal methods could generate realistic longitudinal disease trajectories capturing flare–remission dynamics [30, 32, 33].

### Privacy and regulatory landscape

Differential privacy guarantees have been combined with GAN-based and diffusion-based generation to provide mathematically bounded re-identification protection [29, 46–48], though the relationship between epsilon values and downstream model performance remains context-dependent. Federated learning keeps raw data within institutional boundaries while enabling collaborative training [49–51] and distributed conditional GAN frameworks enable multi-institutional generation in federated settings [52, 53].

The regulatory landscape is complex and unsettled. Within the EU, synthetic data have been proposed as a GDPR compliance pathway, since it may fall outside the scope of personal data protection. However, the legal status remains contested: if the generation process preserves enough statistical detail to enable re-identification, synthetic output might itself constitute personal data [40, 41, 43–45, 54, 55]. Under HIPAA, synthetic data that cannot be linked to real individuals fall outside protected health information, though the generation process must comply with privacy regulations. The EHDS is exploring synthetic data for cross-border secondary use [56, 57].

Regulatory agencies are engaging cautiously. The US Food and Drug Administration’s Digital Health Center of Excellence has explored the use of synthetic data as supplementary evidence in device submissions [58], and the European Medicines Agency recognizes in silico approaches as supportive evidence [41]. Synthetic control arms are being explored for low-prevalence conditions where large, randomized trials are infeasible [9, 10, 59, 60]. QSP-based virtual populations have already enabled RA drug development decisions [9, 61], providing a concrete precedent for regulatory acceptance in rheumatology. Establishing internationally harmonized guidance on synthetic health data classification is a practical precondition for realizing the field’s translational potential.

## Discussion

The findings of this review reveal a field that is technically vibrant but methodologically immature and whose translation to rheumatology demands attention to several intrinsic limitations of synthetic data that are frequently underappreciated in the literature. Despite the rapid growth documented in this review, the majority of included studies remain methodological or proof-of-concept in nature, with relatively few demonstrating clinically validated applications. This is particularly evident in rheumatology, where only 13 of 228 studies address disease-specific questions, highlighting the early translational stage of synthetic data in the field. This underrepresentation is better read as a structural than a conceptual gap: rheumatology adopted machine learning later than image-rich specialties such as radiology and oncology, hosts comparatively few embedded computational research groups and works with datasets that are small, fragmented across many low-prevalence conditions and encumbered by legal restrictions on cross-border registry sharing. The longitudinal and multimodal phenotypes that characterise rheumatic disease are also intrinsically harder to model than the cross-sectional, single-modality data on which most generative methods have matured, further slowing uptake in the specialty. Moreover, most studies evaluate synthetic data through machine learning performance metrics rather than clinical validity or causal inference, leaving the question of whether synthetic data can reliably support clinical decision-making largely unanswered.

The most fundamental concern is error propagation and bias amplification. Synthetic data generators are only as good as the data they learn from. Any biases, errors or systematic distortions present in the training data will be faithfully reproduced and potentially amplified in the synthetic output [39, 62, 63]. A registry with incomplete ascertainment of adverse events, for example, will yield synthetic data that similarly underrepresents those events, and models trained on such synthetic data will inherit this blind spot. Pilgram *et al*. [63] showed that hallucinations in synthetic tabular data can materially degrade the performance of prognostic machine learning models, demonstrating that error propagation is not merely a theoretical concern but a measurable source of harm. Teeple *et al*. [64] further demonstrated that synthetic data can perpetuate and even amplify racial and gender biases embedded in original datasets, raising important equity concerns. In rheumatology, where many datasets originate from tertiary referral centres with inherently skewed case mixes, the risk of propagating selection-related biases is particularly acute.

Closely related is Berkson’s paradox, a form of collider bias arising when data are collected from settings where inclusion is conditioned on a variable influenced by other variables [62, 65]. Hospital-based rheumatology registries are a textbook example: patients are included because they were referred to a specialist centre, and referral itself is influenced by disease severity, unusual presentations and comorbidity burden. This selection mechanism can create spurious correlations between variables that are actually independent in the general population. A synthetic data generator trained on such a registry will treat these artifactual associations as genuine patterns and reproduce them in the synthetic output, effectively propagating and codifying the selection bias [65]. Juwara *et al*. [65] demonstrated that synthetic data augmentation can mitigate some forms of covariate bias, but only when the bias is recognized and explicitly addressed in the generation process, an assumption that is rarely met in practice.

More broadly, synthetic data cannot generate information that is not present, at least implicitly, in the training data [39, 66, 67]. A generator trained on a dataset that lacks representation of a particular disease phenotype or demographic subgroup cannot produce realistic synthetic examples of that missing category. This means that augmentation cannot overcome ascertainment bias or systematic collection gaps, it can only interpolate within available examples. Nazia *et al*. [66] noted that many studies fail to assess whether synthetic data adequately represent the tails of the real data distribution. Vallevik *et al*. [67] proposed a quality assessment framework that explicitly addresses representational completeness, but adoption of such frameworks remains limited. For rheumatology, where low-prevalence manifestations may be absent from even large registries, this is a fundamental constraint that no amount of computational sophistication can fully overcome.

These considerations also clarify how synthetic data generation differs from the more familiar practice of data augmentation, and why the two are best understood as complementary rather than interchangeable. Augmentation techniques such as oversampling, SMOTE and geometric or temporal transformations are simple, computationally cheap and label-preserving, and they are highly effective for correcting class imbalance in tasks such as detecting rare disease manifestations. However, because they only perturb or interpolate existing records, they cannot introduce genuinely new information, cannot create cases of phenotypes never observed in the source data and cannot by themselves enable privacy-preserving data sharing. Synthetic generation, in contrast, models the joint distribution of the data to produce entirely novel records and can therefore support privacy-preserving registry sharing and the construction of virtual or in silico populations. These advantages come at a cost: generative approaches are considerably more complex and computing power intensive, harder to validate and vulnerable to error propagation, mode collapse and residual re-identification risk. The two strategies are thus complementary, and the appropriate choice depends on the goal, with augmentation suited to correcting imbalance within an existing cohort and synthetic generation suited to sharing data or simulating populations that the original dataset cannot supply.

Temporal distribution shift poses a further challenge for synthetic data generated from historical datasets [34, 39]. Clinical practice in rheumatology evolves continuously: the introduction of new biologic and targeted synthetic therapies, changes in classification criteria and evolving treatment targets mean that patient populations and disease trajectories in historical data may not reflect current clinical reality. Miletic and Sariyar [34] specifically examined synthetic data generation for longitudinal health data, noting that temporal coherence remains one of the most challenging aspects of synthetic data quality. Synthetic data trained on historical registries may therefore generate patients whose treatment patterns and disease courses are anachronistic, limiting the utility of such data for contemporary research questions.

These methodological concerns converge on a broader question that Koul *et al*. [39] have termed ‘synthetic trust’: the degree to which clinicians, researchers and regulators can trust findings derived from synthetic data. Our quality assessment reveals that comprehensive validation is the exception rather than the rule. Only 2 of 13 rheumatology studies formally evaluated privacy, only 1 shared code for reproducibility and only 1 adequately discussed limitations. Notably, formal privacy evaluation was absent in the majority of studies and claims about privacy benefits often exceeded the empirical privacy testing performed. Until the field develops and consistently applies standardized validation protocols, the risk remains that synthetic data will be used with unjustified confidence, leading to conclusions that are plausible but wrong.

### Limitations

This review has several limitations that should be considered when interpreting its findings. The most significant concerns the scope of quality assessment. Formal quality evaluation using our purpose-designed 10-item checklist was applied only to the 13 rheumatology-specific studies from which disease-specific conclusions are drawn. The 10-item checklist was author-derived and has not been formally validated, therefore the resulting quality classifications should be interpreted as a structured descriptive appraisal rather than as estimates derived from a validated risk of bias instrument. No formal risk of bias assessment was performed for the remaining 215 studies included for methodological context. This decision was necessitated by the absence of a validated instrument for computational methodology studies, as confirmed by systematic evaluation of RoB 2 [14], ROBINS-I [15], QUADAS-2 [16], PROBAST [17], PROBAST+AI [18] and QUADAS-AI [19]. The absence of such a tool is itself a notable gap in the methodological literature, one that will need to be addressed as systematic reviews of computational methods become more common. The small number of rheumatology-specific studies (*n* = 13) limits the generalizability of disease-specific conclusions. While these 13 studies demonstrate feasibility across six disease areas, drawing firm conclusions about the state of synthetic data in any individual rheumatic disease is premature. Beyond the genuine scarcity of work in this area, publication bias and underreporting of negative findings may also contribute to this low count. Negative or unsuccessful synthetic data experiments, such as generators that fail to reproduce clinical validity, are less likely to reach publication than positive demonstrations, so the corpus we retrieved is skewed toward successful applications. In addition, preprint and venue effects together with our eligibility criteria, which were restricted to full papers, may further undercount early stage or null-result work. The true volume of rheumatology synthetic data experimentation may therefore be underestimated by the published record.

## Conclusions

Synthetic data generation has evolved into a rapidly maturing field with growing applicability to rheumatology. The 228 included studies demonstrate a robust methodological foundation spanning GANs, diffusion models, LLMs, statistical approaches, digital twin frameworks and hybrid methods. Validation frameworks for fidelity, utility and privacy are established in principle but inconsistently applied. The absence of a validated risk of bias tool for this study type is itself indicative of the field’s methodological immaturity.

The 13 rheumatology-specific studies demonstrate feasibility across six diseases. The most advanced work on digital twins [11], virtual populations [9, 10] and privacy-preserving imaging [28] provides proof of concept, yet quality assessment reveals systematic weaknesses in privacy evaluation, reproducibility and limitation reporting.

Looking forward, several priorities emerge as critical for the advancement of synthetic data in rheumatology. The application of validated synthetic data methods to low-prevalence rheumatic diseases, including myositis, vasculitis and scleroderma, where data scarcity most severely limits research, represents perhaps the most immediate opportunity. Equally important is the development of rheumatology-specific validation criteria that go beyond statistical fidelity to incorporate clinical plausibility and expert assessment. Regulatory clarity on the legal status of synthetic health data under GDPR and other frameworks is urgently needed to enable multinational registry sharing. The integration of multimodal synthetic data generation spanning clinical, imaging and genomic modalities would address the inherently multimodal nature of rheumatology research. Finally, the development of a validated risk of bias tool specifically designed for computational methodology studies would strengthen the evidence base not only for synthetic data but for the broader field of AI in medicine.

The convergence of advanced generative modelling, growing regulatory engagement and the pressing unmet needs of rheumatology research creates a window of opportunity. Realizing this potential requires closer collaboration between computational scientists, rheumatologists and regulatory experts and a commitment to rigorous validation, transparent reporting and open science practices that the current literature does not yet consistently demonstrate.

## Supplementary Material

rkag114_Supplementary_Data

## Data Availability

All data supporting the findings of this systematic review are available within the article and its supplementary materials. The complete search strategies, evidence table of all 228 included studies, quality assessment and detailed methods synthesis are provided in the supplementary material. No primary patient-level data were collected for this study. The review protocol is registered on INPLASY (INPLASY202630024; DOI: 10.37766/inplasy2026.3.0024).
