# Supplementary material for: Synthetic data in rheumatology: a systematic literature review
Source: Rheumatol Adv Pract. 2026 Sep 25;10(4):rkag114. doi: 10.1093/rap/rkag114 (PMC13682607; doi:10.1093/rap/rkag114)
Supplement: Supplementary file 1 [file rkag114_supplementary_data.docx]

**Supplementary Materials**

Synthetic Data in Rheumatology:
A Systematic Literature Review

Search period: January 2016 – January 2026

# S1. Complete Search Strategy

## S1.1 Stream A: PubMed/MEDLINE — Rheumatology-Specific

**Database:** PubMed/MEDLINE

**Date range:** January 2016 – January 2026

**Results:** n = 144

**Search query:**

("synthetic data"[tiab] OR "synthetic dataset*"[tiab] OR "synthetic patient*"[tiab] OR "synthetic cohort*"[tiab] OR "synthetic population*"[tiab] OR "digital twin*"[tiab] OR "in silico trial*"[tiab] OR "in-silico trial*"[tiab] OR "virtual population*"[tiab] OR "virtual cohort*"[tiab] OR "data augmentation"[tiab] OR "Computer Simulation"[MeSH])
AND
("Rheumatic Diseases"[MeSH] OR "Arthritis, Rheumatoid"[MeSH] OR "Arthritis"[MeSH] OR "Spondylarthritis"[MeSH] OR "Spondylitis, Ankylosing"[MeSH] OR "Lupus Erythematosus, Systemic"[MeSH] OR "Scleroderma, Systemic"[MeSH] OR "Myositis"[MeSH] OR "Dermatomyositis"[MeSH] OR "Polymyositis"[MeSH] OR "Vasculitis"[MeSH] OR "Sjogren's Syndrome"[MeSH] OR "Gout"[MeSH] OR "Fibromyalgia"[MeSH] OR "Osteoarthritis"[MeSH] OR "Connective Tissue Diseases"[MeSH] OR "Autoimmune Diseases"[MeSH] OR rheumat*[tiab] OR arthritis[tiab] OR spondyloarthrit*[tiab] OR spondylitis[tiab] OR lupus[tiab] OR "systemic sclerosis"[tiab] OR scleroderma[tiab] OR myositis[tiab] OR dermatomyositis[tiab] OR polymyositis[tiab] OR vasculitis[tiab] OR Sjögren*[tiab] OR Sjogren*[tiab] OR gout[tiab] OR "crystal arthropathy"[tiab] OR fibromyalgia[tiab] OR osteoarthritis[tiab] OR "connective tissue disease*"[tiab] OR autoimmun*[tiab])
NOT
("synthetic DMARD*" OR "conventional synthetic" OR "targeted synthetic" OR "cs-DMARD*" OR "ts-DMARD*" OR "csDMARD*" OR "tsDMARD*" OR "b/tsDMARD*")

**Rationale:** The NOT clause excludes papers about synthetic DMARDs (disease-modifying antirheumatic drugs), which constitute the majority of "synthetic" references in rheumatology literature. "Virtual patient" was excluded to avoid matches with medical education contexts; "virtual population" and "virtual cohort" were retained as they specifically relate to pharmacokinetic/pharmacodynamic modeling and in silico trials.

## S1.2 Stream B: PubMed/MEDLINE — Healthcare General

**Database:** PubMed/MEDLINE

**Date range:** January 2016 – January 2026

**Results:** n = 314

**Search query:**

("synthetic data"[ti] OR "synthetic dataset"[ti] OR "synthetic health data"[ti] OR "synthetic EHR"[ti] OR "synthetic electronic health record"[ti] OR "synthetic patient data"[ti] OR "synthetic clinical data"[ti] OR "synthetic medical data"[ti])
AND
("Delivery of Health Care"[MeSH] OR "Electronic Health Records"[MeSH] OR "Medical Informatics"[MeSH] OR "Biomedical Research"[MeSH] OR healthcare[tiab] OR clinical[tiab] OR medical[tiab] OR "health data"[tiab] OR "EHR"[tiab] OR patient[tiab])
NOT
("synthetic DMARD*" OR "conventional synthetic" OR "targeted synthetic")

**Rationale:** Title-restricted search to capture foundational synthetic data methods papers in healthcare without requiring rheumatology-specific disease terms. This complements Stream A by including methodological papers (GANs, privacy frameworks, validation metrics) that are applicable across clinical domains.

## S1.3 Stream C: arXiv — Methods, Privacy, and Digital Twins

**Database:** arXiv (cs.LG, cs.AI, cs.CR, stat.ML, q-bio)

**Date range:** January 2016 – January 2026

**Results:** n = 250 (after internal deduplication of 4 parallel queries)

**Search queries (4 parallel, merged and deduplicated):**

**Query C1:** "synthetic data" AND "healthcare" (97 results)

**Query C2:** "synthetic tabular data" AND "generation" (100 results)

**Query C3:** "digital twin" AND "clinical trial" (20 results)

**Query C4:** "differential privacy" AND "medical data" (33 results)

**Rationale:** PubMed does not index computer science and machine learning conferences (NeurIPS, ICML, AAAI) where foundational methods papers for synthetic data generation (CTGAN, TabDDPM, differential privacy frameworks) are published. arXiv preprints provide access to these methodological contributions.

## S1.4 Stream D: Embase

**Database:** Embase

**Date range:** January 2016 – January 2026

**Results:** n = 391 (all identified as duplicates during cross-database deduplication with PubMed)

**Search query:**

('synthetic data'/de OR 'synthetic data':ti,ab OR 'synthetic dataset*':ti,ab OR 'synthetic patient*':ti,ab OR 'synthetic cohort*':ti,ab OR 'synthetic population*':ti,ab OR 'digital twin*':ti,ab OR 'in silico trial*':ti,ab OR 'virtual population*':ti,ab OR 'virtual cohort*':ti,ab OR 'data augmentation':ti,ab OR 'computer simulation'/exp)
AND
('rheumatic disease'/exp OR 'rheumatoid arthritis'/exp OR 'arthritis'/exp OR 'ankylosing spondylitis'/exp OR 'spondyloarthritis'/exp OR 'systemic lupus erythematosus'/exp OR 'systemic sclerosis'/exp OR 'myositis'/exp OR 'dermatomyositis'/exp OR 'polymyositis'/exp OR 'vasculitis'/exp OR 'Sjogren disease'/exp OR 'gout'/exp OR 'fibromyalgia'/exp OR 'osteoarthritis'/exp OR 'connective tissue disease'/exp OR 'autoimmune disease'/exp OR rheumat*:ti,ab OR arthritis:ti,ab OR spondyloarthrit*:ti,ab OR spondylitis:ti,ab OR lupus:ti,ab OR 'systemic sclerosis':ti,ab OR scleroderma:ti,ab OR myositis:ti,ab OR dermatomyositis:ti,ab OR polymyositis:ti,ab OR vasculitis:ti,ab OR sjogren*:ti,ab OR gout:ti,ab OR fibromyalgia:ti,ab OR osteoarthritis:ti,ab OR autoimmun*:ti,ab)
NOT
('synthetic dmard*' OR 'conventional synthetic' OR 'targeted synthetic' OR 'csdmard*' OR 'tsdmard*')

**Rationale:** Embase search mirroring PubMed strategy with Emtree subject headings in place of MeSH terms. All 391 records were identified as duplicates with PubMed during cross-database deduplication.

## S1.5 Other Methods

**Domain expert identification:** n = 7. Seminal studies identified through reference checking and expert knowledge.

**EU regulatory documents:** Key EU regulatory frameworks (GDPR, AI Act, EHDS, Clinical Trials Regulation, MDR) are cited as primary legal sources in the manuscript but were not screened as study records.

# S2. Relevance Scoring Criteria

All records were scored on a structured 1–10 relevance scale during title and abstract screening. Records scoring 1–4 were excluded. Records scoring 5–10 were retained for eligibility assessment.

| Score Range | Description |
| --- | --- |
| 9–10 | Directly about synthetic data generation, validation, or application in rheumatology or healthcare. Methods papers with clear clinical applicability. |
| 7–8 | Substantive synthetic data methods, digital twin frameworks, or privacy-preserving data sharing applicable to clinical settings. |
| 5–6 | Related methods without clear healthcare application, or basic data augmentation studies that may contribute methodological context. |
| 3–4 | Tangentially related. Mentions synthetic data but primary focus is elsewhere. |
| 1–2 | Not relevant to the review scope. |

# S3. Evidence Table: All 228 Included Studies

The following table lists all 228 studies included in the qualitative synthesis, organized by reference number (alphabetical by first author). Category assignments reflect the primary methodological focus of each study.

| Ref # | First Author (Year) | Title | Category | Rheum |
| --- | --- | --- | --- | --- |
| 1 | Abidoye Itunuoluwa (2025) | Platelets Image Classification Through Data Augmentation: A Comparative Study of | GAN-Based Methods |  |
| 2 | Achterberg Jim (2025) | The Data Sharing Paradox of Synthetic Data in Healthcare. | Privacy-Preserving Methods |  |
| 3 | Achuthan Srisairam (2022) | Leveraging deep learning algorithms for synthetic data generation to design and | LLM-Based Methods |  |
| 4 | Adams Tim (2025) | On the fidelity versus privacy and utility trade-off of synthetic patient data. | Privacy-Preserving Methods |  |
| 5 | Aditi Godbole (2025) | Synthetic Data for Robust AI Model Development in Regulated Enterprises | Reviews & Surveys |  |
| 6 | Akiya Ippei (2024) | Comparison of Synthetic Data Generation Techniques for Control Group Survival Da | GAN-Based Methods |  |
| 7 | Alloza Clara (2023) | A Case for Synthetic Data in Regulatory Decision-Making in Europe. | Rule-Based Simulators |  |
| 8 | Amirsina Torfi (2020) | Differentially Private Synthetic Medical Data Generation using Convolutional GAN | Privacy-Preserving Methods |  |
| 9 | Anantaa Kotal (2024) | KIPPS: Knowledge infusion in Privacy Preserving Synthetic Data Generation | Privacy-Preserving Methods |  |
| 10 | Anantaa Kotal (2025) | Differentially Private Synthetic Data Generation Using Context-Aware GANs | Privacy-Preserving Methods |  |
| 11 | Anjanava Biswas (2024) | Enhancing Clinical Documentation with Synthetic Data: Leveraging Generative Mode | GAN-Based Methods |  |
| 12 | Apellaniz Patricia A (2025) | Advancing Cancer Research with Synthetic Data Generation in Low-Data Scenarios. | Digital Twins / Virtual Patients |  |
| 13 | Arora Anmol (2025) | The urgent need to accelerate synthetic data privacy frameworks for medical rese | Privacy-Preserving Methods |  |
| 14 | Arshia Ilaty (2025) | SynLLM: A Comparative Analysis of Large Language Models for Medical Tabular Synt | LLM-Based Methods |  |
| 15 | Aryan Jadon (2023) | Leveraging Generative AI Models for Synthetic Data Generation in Healthcare: Bal | Reviews & Surveys |  |
| 16 | Assaf Jad F (2024) | Enhancing the Automated Detection of Implantable Collamer Lens Vault Using Gener | Reviews & Surveys |  |
| 17 | Azizi Zahra (2021) | Can synthetic data be a proxy for real clinical trial data? A validation study. | Digital Twins / Virtual Patients |  |
| 18 | Azizi Zahra (2023) | A comparison of synthetic data generation and federated analysis for enabling in | Privacy-Preserving Methods |  |
| 19 | Basri Mohammad Ahmed (2025) | How Useful Is Synthetic Data in Developing Predictive Models for Health? | Validation Frameworks |  |
| 20 | Bedathuru Tanya (2024) | Multiscale, mechanistic model of Rheumatoid Arthritis to enable decision making | Digital Twins / Virtual Patients | Yes |
| 21 | Bhishma Dedhia (2022) | SCouT: Synthetic Counterfactuals via Spatiotemporal Transformers for Actionable | Hybrid Methods |  |
| 22 | Birger Moell (2025) | Voice Cloning for Dysarthric Speech Synthesis: Addressing Data Scarcity in Speec | Healthcare Applications |  |
| 23 | Brekke Pål H (2021) | Synthetic data for annotation and extraction of family history information from | Reviews & Surveys |  |
| 24 | Brian Belgodere (2023) | Auditing and Generating Synthetic Data with Controllable Trust Trade-offs | Validation Frameworks |  |
| 25 | Burgess Mark A (2025) | Privacy-hardened and hallucination-resistant synthetic data generation with logi | Privacy-Preserving Methods |  |
| 26 | Chaithra Umesh (2025) | Dependency-aware synthetic tabular data generation | Hybrid Methods |  |
| 27 | Chao Pang (2024) | CEHR-GPT: Generating Electronic Health Records with Chronological Patient Timeli | LLM-Based Methods |  |
| 28 | Chen Junqiao (2019) | The validity of synthetic clinical data: a validation study of a leading synthet | LLM-Based Methods |  |
| 29 | Chen Xingran (2025) | Generating synthetic electronic health record data: a methodological scoping rev | Statistical Methods |  |
| 30 | Cheng Yougan (2022) | Virtual Populations for Quantitative Systems Pharmacology Models. | Digital Twins / Virtual Patients |  |
| 31 | Chhoa Henri (2023) | Improvement of an External Predictive Model Based on New Information Using a Syn | Privacy-Preserving Methods |  |
| 32 | Cogan N G (2021) | Data assimilation of synthetic data as a novel strategy for predicting disease p | Digital Twins / Virtual Patients |  |
| 33 | Coleman Laura Jane (2025) | Patient-Specific Variability in Interleukin-6 and Myeloperoxidase Responses in O | GAN-Based Methods | Yes |
| 34 | Cruz-Parada Elí (2026) | Proof-of-Concept Machine Learning Framework for Arboviral Disease Classification | Rule-Based Simulators |  |
| 35 | D'Amico Saverio (2023) | Synthetic Data Generation by Artificial Intelligence to Accelerate Research and | GAN-Based Methods |  |
| 36 | Daniel McDuff (2023) | Synthetic Data in Healthcare | Reviews & Surveys |  |
| 37 | Daniele Bertolini (2020) | Modeling Disease Progression in Mild Cognitive Impairment and Alzheimer's Diseas | Digital Twins / Virtual Patients |  |
| 38 | Danilov Viacheslav V (2023) | Use of semi-synthetic data for catheter segmentation improvement. | Digital Twins / Virtual Patients |  |
| 39 | Davalan William (2025) | Reproduction of Original Glioblastoma and Brain Metastasis Research Findings Usi | Validation Frameworks |  |
| 40 | David Fuentes (2023) | Distributed Conditional GAN (discGAN) For Synthetic Healthcare Data Generation | GAN-Based Methods |  |
| 41 | Debnath Minakshi (2025) | Enhancing Wearable Fall Detection System via Synthetic Data. | Rule-Based Simulators |  |
| 42 | Demirel Mehmet (2025) | EmiNet: Moving bacteria detection on optical endomicroscopy images trained on sy | Rule-Based Simulators |  |
| 43 | Dinh Thu (2025) | Evaluating Privacy and Utility in Synthetic EHR Data Generation for Adverse Drug | Digital Twins / Virtual Patients |  |
| 44 | Doste Ruben (2022) | Training machine learning models with synthetic data improves the prediction of | Reviews & Surveys |  |
| 45 | Draghi Barbara (2024) | Identifying and handling data bias within primary healthcare data using syntheti | LLM-Based Methods |  |
| 46 | Ebert Nikolas (2025) | Enhancing robustness and generalization in microbiological few-shot detection th | Diffusion Models |  |
| 47 | El Emam Khaled (2020) | Evaluating Identity Disclosure Risk in Fully Synthetic Health Data: Model Develo | Privacy-Preserving Methods |  |
| 48 | El Emam Khaled (2022) | Validating a membership disclosure metric for synthetic health data. | Validation Frameworks |  |
| 49 | El Emam Khaled (2022) | Utility Metrics for Evaluating Synthetic Health Data Generation Methods: Validat | Validation Frameworks |  |
| 50 | Elvatun Severin (2025) | Synthetic data as external control arms in scarce single-arm clinical trials. | Digital Twins / Virtual Patients |  |
| 51 | Ethan Schonfeld (2022) | Generative Adversarial Network Based Synthetic Learning and a Novel Domain Relev | GAN-Based Methods |  |
| 52 | Eyal German (2025) | MIA-EPT: Membership Inference Attack via Error Prediction for Tabular Data | Privacy-Preserving Methods |  |
| 53 | Farhadyar Kiana (2024) | Combining propensity score methods with variational autoencoders for generating | Hybrid Methods |  |
| 54 | Finetti Rebecca (2025) | Data Augmentation and Synthetic Data Generation in Rare Disease Research: A Scop | Reviews & Surveys |  |
| 55 | Florent Guépin (2023) | Synthetic is all you need: removing the auxiliary data assumption for membership | Privacy-Preserving Methods |  |
| 56 | Fok Wai Yan Ryana (2024) | Adversarial robustness improvement for X-ray bone segmentation using synthetic d | Reviews & Surveys |  |
| 57 | Foppa Caterina (2025) | Synthetic Data Generated by Artificial Intelligence to Optimize Surgical Trial D | Validation Frameworks |  |
| 58 | Frayling Lora (2025) | A review of synthetic data terminology for privacy preserving use cases. | Reviews & Surveys |  |
| 59 | Freise Nina (2025) | The Role of Domain-Specific Models for Synthetic Data Generation with Iterative | LLM-Based Methods |  |
| 60 | Gallos Parisis (2024) | INSAFEDARE Project: Innovative Applications of Assessment and Assurance of Data | Statistical Methods |  |
| 61 | Gao Cong (2023) | Synthetic data accelerates the development of generalizable learning-based algor | Hybrid Methods |  |
| 62 | Garbey Marc (2025) | Bridging the Heterogeneity of Myasthenia Gravis Severity Scores for Digital Twin | Digital Twins / Virtual Patients |  |
| 63 | Garbey Marc (2025) | Bridging the Heterogeneity of Myasthenia Gravis Scores as a Foundational Step To | Digital Twins / Virtual Patients |  |
| 64 | Garg Muskan (2025) | Leveraging Reddit data for Context-enhanced Synthetic Health Data Generation to | LLM-Based Methods |  |
| 65 | Georgiou Georgios P (2025) | Enhancing Developmental Language Disorder Identification with Artificial Intelli | Digital Twins / Virtual Patients |  |
| 66 | Giorgio Visani (2022) | Enabling Synthetic Data adoption in regulated domains | Validation Frameworks |  |
| 67 | Giouroukou Konstantina (2026) | Rethinking Privacy in Medical Imaging AI: From Metadata and Pixel-Level Identifi | Privacy-Preserving Methods |  |
| 68 | Goncalves Andre (2020) | Generation and evaluation of synthetic patient data. | LLM-Based Methods |  |
| 69 | Greenberg Jacob K (2023) | Leveraging Artificial Intelligence and Synthetic Data Derivatives for Spine Surg | Reviews & Surveys |  |
| 70 | Grössler Michael (2025) | Open-Source Synthetic Data Generation of Clinical Routine Data. | Hybrid Methods |  |
| 71 | Guanglin Zhou (2025) | Generating Clinically Realistic EHR Data via a Hierarchy- and Semantics-Guided T | Hybrid Methods |  |
| 72 | Guillaudeux Morgan (2023) | Patient-centric synthetic data generation, no reason to risk re-identification i | Privacy-Preserving Methods |  |
| 73 | Guo Aixia (2020) | The Use of Synthetic Electronic Health Record Data and Deep Learning to Improve | Privacy-Preserving Methods |  |
| 74 | Guruge Kasunika (2025) | MetaAcuPoint: MetaHuman-Generated Synthetic Data for Hand Acupoint Localization. | Rule-Based Simulators |  |
| 75 | Gutsche Annika (2025) | Can Synthetic Data Allow for Smaller Sample Sizes in Chronic Urticaria Research? | Statistical Methods |  |
| 76 | Haddad Tia (2025) | Design and Development of SPADE: A Synthetic Patient Data Generator for Testing | Rule-Based Simulators |  |
| 77 | Hahn Waldemar (2022) | Contribution of Synthetic Data Generation towards an Improved Patient Stratifica | Statistical Methods |  |
| 78 | Hahn Waldemar (2024) | Synthetic Data Generation in Hematology - Paving the Way for OMOP and FHIR Integ | Healthcare Applications |  |
| 79 | Hao Jinkui (2026) | Coronary artery segmentation in non-contrast cardiac CT using anatomy-informed c | Hybrid Methods |  |
| 80 | Hugo Chenel (2024) | Multilayer network approaches to omics data integration in Digital Twins for can | Digital Twins / Virtual Patients |  |
| 81 | Hwang Elizabeth E (2025) | Deep Learning Detection of Retinitis Pigmentosa Inheritance Forms through Synthe | Hybrid Methods |  |
| 82 | Iyer A Anandita (2025) | Design and analysis of TwinCardio framework to detect and monitor cardiovascular | Digital Twins / Virtual Patients |  |
| 83 | Jacobs Flavia (2023) | Opportunities and Challenges of Synthetic Data Generation in Oncology. | Reviews & Surveys |  |
| 84 | James Jordon (2020) | Synthetic Data: Opening the data floodgates to enable faster, more directed deve | Reviews & Surveys |  |
| 85 | Jayakumar Prakash (2025) | Shared decision making using digital twins in knee osteoarthritis care: a random | Digital Twins / Virtual Patients | Yes |
| 86 | Jeanson Francis (2024) | Medical calculators derived synthetic cohorts: a novel method for generating syn | Privacy-Preserving Methods |  |
| 87 | Ji Eunjeong (2025) | Evaluating the utility of data integration with synthetic data and statistical m | Validation Frameworks |  |
| 88 | Jia Jingnan (2024) | Explainable fully automated CT scoring of interstitial lung disease for patients | Healthcare Applications | Yes |
| 89 | Jiang Bei (2022) | Balancing Inferential Integrity and Disclosure Risk via Model Targeted Masking a | Privacy-Preserving Methods | Yes |
| 90 | Jiang Yulun (2024) | Generative models for synthetic data generation: application to pharmacokinetic/ | Digital Twins / Virtual Patients |  |
| 91 | Johnson Luke G (2025) | A biomechanical digital twin of Legg-Calvé-Perthes disease deformity. | Digital Twins / Virtual Patients |  |
| 92 | Jonathan R. Walsh (2020) | Generating Digital Twins with Multiple Sclerosis Using Probabilistic Neural Netw | Digital Twins / Virtual Patients |  |
| 93 | Julia Camps (2023) | Digital Twinning of the Human Ventricular Activation Sequence to Clinical 12-lea | Digital Twins / Virtual Patients |  |
| 94 | Julia Camps (2024) | Cardiac Digital Twin Pipeline for Virtual Therapy Evaluation | Digital Twins / Virtual Patients |  |
| 95 | Juwara Lamin (2024) | An evaluation of synthetic data augmentation for mitigating covariate bias in he | Privacy-Preserving Methods |  |
| 96 | Kaabachi Bayrem (2022) | Generation and Evaluation of Synthetic Data in a University Hospital Setting. | Reviews & Surveys |  |
| 97 | Kaabachi Bayrem (2025) | A scoping review of privacy and utility metrics in medical synthetic data. | Privacy-Preserving Methods |  |
| 98 | Kafatos George (2025) | Leveraging Synthetic Data to Facilitate Research: A Collaborative Model for Anal | Privacy-Preserving Methods |  |
| 99 | Karimian Sichani Elnaz (2024) | Creating High-Quality Synthetic Health Data: Framework for Model Development and | Statistical Methods |  |
| 100 | Ke Yu (2025) | SHAP Distance: An Explainability-Aware Metric for Evaluating the Semantic Fideli | Validation Frameworks |  |
| 101 | Keira Behal (2023) | MCRAGE: Synthetic Healthcare Data for Fairness | Diffusion Models |  |
| 102 | Kenta Oono (2023) | Virtual Human Generative Model: Masked Modeling Approach for Learning Human Char | Hybrid Methods |  |
| 103 | Keying Kuang (2024) | Med-Real2Sim: Non-Invasive Medical Digital Twins using Physics-Informed Self-Sup | Digital Twins / Virtual Patients |  |
| 104 | Khartik Uppalapati (2025) | RareGraph-Synth: Knowledge-Guided Diffusion Models for Generating Privacy-Preser | Diffusion Models |  |
| 105 | Khosravi Bardia (2024) | Synthetically enhanced: unveiling synthetic data's potential in medical imaging | Reviews & Surveys |  |
| 106 | Kim Jinah (2024) | Development of a cerebellar ataxia diagnosis model using conditional GAN-based s | GAN-Based Methods |  |
| 107 | Kong Hyoun-Joong (2022) | Automation of generative adversarial network-based synthetic data-augmentation f | Diffusion Models |  |
| 108 | Koul Arman (2025) | Synthetic data, synthetic trust: navigating data challenges in the digital revol | Validation Frameworks |  |
| 109 | Kumari Chandrani (2024) | MMM and MMMSynth: Clustering of heterogeneous tabular data, and synthetic data g | Statistical Methods |  |
| 110 | Kuo Nicholas I-Hsien (2023) | Generating synthetic clinical data that capture class imbalanced distributions w | GAN-Based Methods |  |
| 111 | Kühnel Lisa (2024) | Synthetic data generation for a longitudinal cohort study - evaluation, method e | Validation Frameworks |  |
| 112 | Lacasa Marcos (2023) | A synthetic data generation system for myalgic encephalomyelitis/chronic fatigue | GAN-Based Methods |  |
| 113 | Lai Tim C (2025) | Roles of Artificial Intelligence--Based Synthetic Data in Health Economics and O | Digital Twins / Virtual Patients |  |
| 114 | LeBien Jack (2026) | RINet: synthetic data training for indirect estimation of clinical reference dis | Statistical Methods |  |
| 115 | Lee Yun Gyeong (2025) | Synthetic data production for biomedical research. | Rule-Based Simulators |  |
| 116 | Liu Ruhan (2023) | TMM-Nets: Transferred Multi- to Mono-Modal Generation for Lupus Retinopathy Diag | GAN-Based Methods | Yes |
| 117 | Liu Yintong (2025) | Preserving privacy in healthcare: A systematic review of deep learning approache | Reviews & Surveys |  |
| 118 | Lorge Isabelle (2025) | Detecting the clinical features of difficult-to-treat depression using synthetic | LLM-Based Methods |  |
| 119 | Lun Ronda (2024) | Synthetic data in cancer and cerebrovascular disease research: A novel approach | Privacy-Preserving Methods |  |
| 120 | Mahesh Vaijainthymala Krishnamoorthy (2024) | Data Obfuscation through Latent Space Projection (LSP) for Privacy-Preserving AI | Privacy-Preserving Methods |  |
| 121 | Mahmoud Ahsanullah Yunas (2023) | Early diagnosis and personalised treatment focusing on synthetic data modelling: | GAN-Based Methods |  |
| 122 | Manjunath Mahendra (2024) | Convex space learning for tabular synthetic data generation | GAN-Based Methods |  |
| 123 | Manuel Cossio (2022) | A perspective on the use of health digital twins in computational pathology | Digital Twins / Virtual Patients |  |
| 124 | Marchesi Raffaele (2025) | Generative AI mitigates representation bias and improves model fairness through | Reviews & Surveys |  |
| 125 | Margaux Tornqvist (2024) | A text-to-tabular approach to generate synthetic patient data using LLMs | LLM-Based Methods |  |
| 126 | Marino Simeone (2025) | Medical data sharing and synthetic clinical data generation - maximizing biomedi | Digital Twins / Virtual Patients |  |
| 127 | Mezzalira Elisabetta (2025) | Synthetic data generation in paediatrics and paediatric nursing: what, how, and | Reviews & Surveys |  |
| 128 | Miletic Marko (2025) | Synthetic data generation methods for longitudinal and time series health data: | Reviews & Surveys |  |
| 129 | Miletic Marko (2025) | Synthetic data for pharmacogenetics: enabling scalable and secure research. | Validation Frameworks |  |
| 130 | Miletic Marko (2025) | Utility-based Analysis of Statistical Approaches and Deep Learning Models for Sy | Validation Frameworks |  |
| 131 | Mischa Dombrowski (2024) | Uncovering Hidden Subspaces in Video Diffusion Models Using Re-Identification | Privacy-Preserving Methods |  |
| 132 | Moazemi Sobhan (2024) | NFDI4Health Workflow and Service for Synthetic Data Generation, Assessment and R | Privacy-Preserving Methods |  |
| 133 | Mohri Ohmi (2025) | Evaluation of Synthetic Data Generation Methods for Medical Tabular Data: Repres | Validation Frameworks |  |
| 134 | Moroianu Stefania L (2025) | Improving Performance, Robustness, and Fairness of Radiographic AI Models with F | Diffusion Models |  |
| 135 | Moya-Sáez Elisa (2021) | A deep learning approach for synthetic MRI based on two routine sequences and tr | GAN-Based Methods |  |
| 136 | Mwigereri Dorcas G (2025) | Synthetic data generation of health and demographic surveillance systems data: a | GAN-Based Methods |  |
| 137 | Nagesh Nitish (2025) | FairCauseSyn: Towards Causally Fair LLM-augmented Synthetic Data Generation. | LLM-Based Methods |  |
| 138 | Nameyeh Alam (2024) | Digital Twin Generators for Disease Modeling | Digital Twins / Virtual Patients |  |
| 139 | Navid Ashrafi (2024) | Protect and Extend -- Using GANs for Synthetic Data Generation of Time-Series Me | GAN-Based Methods |  |
| 140 | Nazia Nafis (2025) | Critical Challenges and Guidelines in Evaluating Synthetic Tabular Data: A Syste | Reviews & Surveys |  |
| 141 | Niki Z. Petrakos (2025) | A Framework for Generating Realistic Synthetic Tabular Data in a Randomized Cont | Hybrid Methods |  |
| 142 | Ofer Mendelevitch (2021) | Fidelity and Privacy of Synthetic Medical Data | Reviews & Surveys |  |
| 143 | Ogwel Billy (2025) | A quarter-century of synthetic data in healthcare: Unveiling trends with structu | Reviews & Surveys |  |
| 144 | Padoan Andrea (2024) | Dynamic mirroring: unveiling the role of digital twins, artificial intelligence | Digital Twins / Virtual Patients |  |
| 145 | Pallavi Bhardwaj (2025) | TabularQGAN: A Quantum Generative Model for Tabular Data | GAN-Based Methods |  |
| 146 | Pandita Aakriti (2025) | Synthetic data trained open-source language models are feasible alternatives to | LLM-Based Methods |  |
| 147 | Parise Orlando (2025) | CTGAN-driven synthetic data generation: A multidisciplinary, expert-guided appro | GAN-Based Methods |  |
| 148 | Patricia A. Apellániz (2024) | An improved tabular data generator with VAE-GMM integration | Hybrid Methods |  |
| 149 | Paul Nathalie (2024) | Building virtual patients using simulation-based inference. | Digital Twins / Virtual Patients | Yes |
| 150 | Pesapane Filippo (2025) | The Role of Synthetic Data and Generative AI in Breast Imaging: Promise, Pitfall | Reviews & Surveys |  |
| 151 | Pezoulas Vasileios C (2021) | A computational pipeline for data augmentation towards the improvement of diseas | Statistical Methods | Yes |
| 152 | Pezoulas Vasileios C (2022) | Bayesian Inference-Based Gaussian Mixture Models With Optimal Components Estimat | Reviews & Surveys |  |
| 153 | Pezoulas Vasileios C (2023) | Boosting the performance of MALT lymphoma classification in patients with primar | Statistical Methods | Yes |
| 154 | Pezoulas Vasileios C (2025) | Utilizing synthetic data for privacy-preserving AI modeling in radiomics: a case | Digital Twins / Virtual Patients |  |
| 155 | Pilgram Lisa (2025) | Protecting patient privacy in tabular synthetic health data: a regulatory perspe | Privacy-Preserving Methods |  |
| 156 | Pilgram Lisa (2025) | Magnitude and Impact of Hallucinations in Tabular Synthetic Health Data on Progn | Validation Frameworks |  |
| 157 | Polycarp Nalela (2025) | Leveraging Generative AI Through Prompt Engineering and Rigorous Validation to C | LLM-Based Methods |  |
| 158 | Pozzi Matteo (2024) | Generating and evaluating synthetic data in digital pathology through diffusion | Reviews & Surveys |  |
| 159 | Prasanna Anish (2023) | Synthetic Health Data Can Augment Community Research Efforts to Better Inform th | Validation Frameworks |  |
| 160 | Prediger Lukas (2024) | Collaborative learning from distributed data with differentially private synthet | Privacy-Preserving Methods |  |
| 161 | Prezja Fabi (2022) | DeepFake knee osteoarthritis X-rays from generative adversarial neural networks | GAN-Based Methods | Yes |
| 162 | Pstras Leszek (2025) | A Synthetic Dataset for in Silico Studies of Parameters Influencing Changes in M | Digital Twins / Virtual Patients |  |
| 163 | Qian Zhaozhi (2024) | Synthetic data for privacy-preserving clinical risk prediction. | Diffusion Models |  |
| 164 | Rafiei Alireza (2023) | Improving irregular temporal modeling by integrating synthetic data to the elect | GAN-Based Methods |  |
| 165 | Rajotte Jean-Francois (2022) | Synthetic data as an enabler for machine learning applications in medicine. | Healthcare Applications |  |
| 166 | Reiner Benaim Anat (2020) | Analyzing Medical Research Results Based on Synthetic Data and Their Relation to | Privacy-Preserving Methods |  |
| 167 | Resmi Ramachandranpillai (2024) | Bt-GAN: Generating Fair Synthetic Healthdata via Bias-transforming Generative Ad | GAN-Based Methods |  |
| 168 | Resnick Dean M (2021) | Using Synthetic Data to Replace Linkage Derived Elements: A Case Study. | Privacy-Preserving Methods |  |
| 169 | Rodriguez-Almeida Antonio J (2023) | Synthetic Patient Data Generation and Evaluation in Disease Prediction Using Sma | Statistical Methods |  |
| 170 | Ruixiang Tang (2023) | Does Synthetic Data Generation of LLMs Help Clinical Text Mining? | LLM-Based Methods |  |
| 171 | Rujas Miguel (2025) | Synthetic data generation in healthcare: A scoping review of reviews on domains, | Healthcare Applications |  |
| 172 | Rumeng Li (2025) | DualAlign: Generating Clinically Grounded Synthetic Data | LLM-Based Methods |  |
| 173 | S. M. Mustaqim (2025) | When Privacy Isn't Synthetic: Hidden Data Leakage in Generative AI Models | Privacy-Preserving Methods |  |
| 174 | Santangelo Gabriele (2025) | How good is your synthetic data? SynthRO, a dashboard to evaluate and benchmark | Validation Frameworks |  |
| 175 | Schaudt Daniel (2023) | A Critical Assessment of Generative Models for Synthetic Data Augmentation on Li | Validation Frameworks |  |
| 176 | Schulz Nicolas Alexander (2024) | Learning debiased graph representations from the OMOP common data model for synt | Hybrid Methods |  |
| 177 | Scroggins Jihye Kim (2025) | Does synthetic data augmentation improve the performances of machine learning cl | GAN-Based Methods |  |
| 178 | Segal Bradley (2025) | Bridging the Generalisation Gap: Synthetic Data Generation for Multi-Site Clinic | Rule-Based Simulators |  |
| 179 | Shi Jingpu (2022) | Generating high-fidelity privacy-conscious synthetic patient data for causal eff | Reviews & Surveys |  |
| 180 | Sina Saffaran (2025) | Digital Twins of Mechanically Ventilated Preterm Neonates with Respiratory Distr | Digital Twins / Virtual Patients |  |
| 181 | Siyeop Yoon (2025) | Cascaded 3D Diffusion Models for Whole-body 3D 18-F FDG PET/CT synthesis from De | Diffusion Models |  |
| 182 | Smolyak Daniel (2024) | Large language models and synthetic health data: progress and prospects. | Reviews & Surveys |  |
| 183 | Stefano Sinisi (2021) | Optimal personalised treatment computation through in silico clinical trials on | Digital Twins / Virtual Patients |  |
| 184 | Steven Bedrick (2025) | A Typology of Synthetic Datasets for Dialogue Processing in Clinical Contexts | Reviews & Surveys |  |
| 185 | Suhas BN (2025) | How Real Are Synthetic Therapy Conversations? Evaluating Fidelity in Prolonged E | Validation Frameworks |  |
| 186 | Sun Hanxi (2023) | A deep learning approach to private data sharing of medical images using conditi | GAN-Based Methods | Yes |
| 187 | Suresh Krish (2023) | Generation of synthetic tympanic membrane images: Development, human validation, | Validation Frameworks |  |
| 188 | Susser Daniel (2024) | Synthetic Health Data: Real Ethical Promise and Peril. | Validation Frameworks |  |
| 189 | Syed Hasib Akhter Faruqui (2024) | Nurse-in-the-Loop Artificial Intelligence for Precision Management of Type 2 Dia | Digital Twins / Virtual Patients |  |
| 190 | Tagmatova Zarnigor (2023) | New Approach for Generating Synthetic Medical Data to Predict Type 2 Diabetes. | GAN-Based Methods |  |
| 191 | Teeple Stephanie (2025) | Synthetic data and health equity: accounting for racism and sexism in health car | Validation Frameworks |  |
| 192 | Tian Muhang (2024) | Reliable generation of privacy-preserving synthetic electronic health record tim | Reviews & Surveys |  |
| 193 | Tobias Hyrup (2023) | Sharing is CAIRing: Characterizing Principles and Assessing Properties of Univer | Privacy-Preserving Methods |  |
| 194 | Tomohiro Kikuchi (2023) | Method for Generating Synthetic Data Combining Chest Radiography Images with Tab | GAN-Based Methods |  |
| 195 | Torres-Silva Ever Augusto (2025) | Synthetic data within a common data model for artificial intelligence applicatio | Rule-Based Simulators |  |
| 196 | Touati Hanae (2025) | AI-driven approach for creating and evaluating a synthetic dataset for Medicatio | LLM-Based Methods |  |
| 197 | Trisha Das (2024) | Synthetic Patient-Physician Dialogue Generation from Clinical Notes Using LLM | LLM-Based Methods |  |
| 198 | Tsao Shu-Feng (2023) | Health Synthetic Data to Enable Health Learning System and Innovation: A Scoping | Reviews & Surveys |  |
| 199 | Tu Lorna (2025) | An approach for cancer outcomes modelling using a comprehensive synthetic datase | GAN-Based Methods |  |
| 200 | Tucker Allan (2020) | Generating high-fidelity synthetic patient data for assessing machine learning h | Reviews & Surveys |  |
| 201 | Valerio Guarrasi (2025) | Whole-Body Image-to-Image Translation for a Virtual Scanner in a Healthcare Digi | Digital Twins / Virtual Patients |  |
| 202 | van Dijk Bram (2024) | A Novel Taxonomy for Navigating and Classifying Synthetic Data in Healthcare App | Statistical Methods |  |
| 203 | Vibeke Binz Vallevik (2024) | Can I trust my fake data -- A comprehensive quality assessment framework for syn | Validation Frameworks |  |
| 204 | Vilardell Mireia (2020) | Missing data imputation and synthetic data simulation through modeling graphical | Statistical Methods |  |
| 205 | Walonoski Jason (2020) | Synthea™ Novel coronavirus (COVID-19) model and synthetic data set. | Rule-Based Simulators |  |
| 206 | Wang Echo (2024) | Validation Assessment of Privacy-Preserving Synthetic Electronic Health Record D | Privacy-Preserving Methods |  |
| 207 | Wang Echo H (2025) | Crossing borders securely: synthetic data and federated networks for privacy-pre | Privacy-Preserving Methods |  |
| 208 | Wiedekopf Joshua (2021) | Desiderata for a Synthetic Clinical Data Generator. | Rule-Based Simulators |  |
| 209 | Williams Michelle C (2025) | New ways to use imaging data in cardiovascular research: survey of opinions on f | Healthcare Applications |  |
| 210 | Woillard Jean-Baptiste (2025) | To be or not to be, when synthetic data meet clinical pharmacology: A focused st | Validation Frameworks |  |
| 211 | Xiaodan Xing (2022) | HDL: Hybrid Deep Learning for the Synthesis of Myocardial Velocity Maps in Digit | Digital Twins / Virtual Patients |  |
| 212 | Xie Qiaoqiao (2024) | Prediction of Treatment Effect of SLE-ITP Patients Based on Cost-Sensitive Neura | Hybrid Methods | Yes |
| 213 | Xu Jingxi (2025) | ChatEMG: Synthetic Data Generation to Control a Robotic Hand Orthosis for Stroke | LLM-Based Methods |  |
| 214 | Yan Chao (2022) | A Multifaceted benchmarking of synthetic electronic health record generation mod | Reviews & Surveys |  |
| 215 | Yan Chao (2024) | Generating Synthetic Electronic Health Record Data Using Generative Adversarial | Diffusion Models |  |
| 216 | Yang Hong (2019) | Synthetic data framework to estimate the minimum detectable concentration of con | Hybrid Methods |  |
| 217 | Yaobin Ling (2024) | MALLM-GAN: Multi-Agent Large Language Model as Generative Adversarial Network fo | LLM-Based Methods |  |
| 218 | Yihan Lin (2025) | A Case Study Exploring the Current Landscape of Synthetic Medical Record Generat | LLM-Based Methods |  |
| 219 | Yue Wang (2024) | TWIN-GPT: Digital Twins for Clinical Trials via Large Language Model | Digital Twins / Virtual Patients |  |
| 220 | Zamzmi Ghada (2025) | Scorecard for synthetic medical data evaluation. | Validation Frameworks |  |
| 221 | Zazzetti Elena (2025) | Longitudinal Synthetic Data Generation by Artificial Intelligence to Accelerate | Hybrid Methods |  |
| 222 | Zerrouk Naouel (2024) | Building a modular and multi-cellular virtual twin of the synovial joint in Rheu | Digital Twins / Virtual Patients | Yes |
| 223 | Zhang Jing (2026) | Semi-supervised learning for dose prediction in targeted radionuclide therapy: a | Hybrid Methods |  |
| 224 | Zhang Yili (2024) | Exploring the Utilization of Synthetic Data in Unsupervised Clustering for Opioi | GAN-Based Methods |  |
| 225 | Zheng Huimin (2025) | Profile-aided distillation framework for personalized sleep analysis with compac | LLM-Based Methods |  |
| 226 | Zhou Nina (2022) | DataSifter II: Partially synthetic data sharing of sensitive information contain | Digital Twins / Virtual Patients |  |
| 227 | Zifeng Wang (2022) | PromptEHR: Conditional Electronic Healthcare Records Generation with Prompt Lear | LLM-Based Methods |  |
| 228 | Zolnour Ali (2025) | LLMCARE: early detection of cognitive impairment via transformer models enhanced | LLM-Based Methods |  |

# S4. Study Category Definitions

**Digital Twins / Virtual Patients (n=37)**

Studies focused on computational models of individual patients or virtual patient populations for in silico clinical trials, drug development simulation, or personalized treatment optimization.

**Privacy-Preserving Methods (n=32)**

Studies primarily addressing privacy evaluation, disclosure risk, membership inference attacks, differential privacy, or federated learning in the context of synthetic health data.

**Reviews & Surveys (n=31)**

Systematic reviews, scoping reviews, narrative reviews, and survey papers on synthetic data in healthcare, including terminology frameworks and ethical analyses.

**GAN-Based Methods (n=27)**

Studies developing or evaluating generative adversarial network architectures for synthetic health data generation, including CTGAN, WGAN, conditional GAN, and GAN-based imaging synthesis.

**Validation Frameworks (n=25)**

Studies focused on metrics, benchmarks, and evaluation frameworks for assessing synthetic data quality across fidelity, utility, and privacy dimensions.

**LLM-Based Methods (n=23)**

Studies using large language models for synthetic health data generation, including clinical text, EHR records, patient-physician dialogues, and prompt-based tabular data.

**Hybrid Methods (n=16)**

Studies combining multiple generative paradigms (e.g., VAE-GAN, physics-informed neural networks, simulation-based inference with QSP models, multi-modal synthesis).

**Statistical Methods (n=12)**

Studies using classical statistical approaches including Bayesian networks, copulas, Gaussian mixture models, and propensity score methods for synthetic data generation.

**Rule-Based Simulators (n=11)**

Studies using rule-based simulation platforms (e.g., Synthea) or domain-specific mechanistic simulators encoding epidemiological and clinical knowledge for synthetic patient generation.

**Diffusion Models (n=8)**

Studies applying denoising diffusion probabilistic models or score-based generative models to tabular, imaging, or time-series healthcare data.

**Healthcare Applications (n=6)**

Studies primarily demonstrating downstream healthcare applications of synthetic data without a primary methodological contribution to data generation.

# S5. Exclusion Criteria Applied at Full-Text Assessment

**E1: Data augmentation as preprocessing (n=62)**

Papers that use synthetic data augmentation (e.g., oversampling, SMOTE, or GAN-generated samples) solely as a preprocessing step to improve a downstream clinical classifier, without contributing to synthetic data generation methodology, validation, or the broader synthetic data discourse.

**E2: Conference abstract or poster only (n=1)**

Conference abstracts, poster presentations, or oral presentation summaries without a full-length paper (fewer than 4 pages of methodological content).

**E3: Commentary, editorial, or letter without original data (n=23)**

Editorials, commentaries, perspectives, viewpoints, and letters to the editor that discuss synthetic data concepts without presenting original data, methods, or results.

**E4: Not about healthcare/clinical data (n=34)**

Papers about synthetic data generation in non-healthcare domains (e.g., finance, autonomous vehicles, computer vision benchmarks) that were indexed due to broad search terms.

**S6. Supplementary Methods: Detailed Synthesis of Generation Methods, Validation Frameworks, and Healthcare Applications**

**Methods for Synthetic Data Generation**

Classical statistical approaches represent the foundation upon which the field was built. Bayesian network-based synthesis encodes conditional dependencies between variables, preserving the complex interrelationships that characterize clinical data [S1-S4]. Copula-based approaches and Gaussian mixture models offer complementary strengths for clinical trial simulation and pharmacogenetics [S5-S10]. The interpretability of these methods remains a significant advantage for regulatory acceptance. Propensity mean squared error (pMSE) has emerged as a useful distinguishability metric [S11,S12].

Deep learning methods now dominate the synthetic data landscape. GANs have been benchmarked extensively through variants such as CTGAN and TVAE across electronic health record datasets [S13,S14], with privacy-aware versions providing differential privacy guarantees [S15]. GAN-based methods span diverse clinical imaging modalities including MRI, CT, X-ray, and histopathology [S16-S38], though they can suffer from mode collapse and training instability. Diffusion models overcome these limitations through iterative denoising, with TabDDPM and conditional denoising diffusion probabilistic models adapted for heterogeneous healthcare data [S39-S41]. They have demonstrated improved fidelity for privacy-preserving synthetic EHR time series [S42-S44] and high-dimensional medical imaging [S45,S46], and knowledge-guided variants have been proposed specifically for low-prevalence disease data generation [S47].

Rule-based simulation platforms such as Synthea generate clinically coherent patient trajectories through explicitly coded clinical knowledge [S48], with domain-specific simulators extending this approach to specialized contexts [S49-S57]. Their strength lies in clinical plausibility by construction, though they cannot discover novel distributional features in real data.

LLMs have introduced a fundamentally new paradigm through semantic understanding and contextual reasoning [S58]. CEHR-GPT generates longitudinal electronic health records with chronological event sequences [S59-S61], and applications span clinical documentation, therapy synthesis, and dialogue generation [S62-S80]. The risk of hallucination requires rigorous validation [S81].

Emerging hybrid methods combine complementary strengths: VAE-GAN architectures merge variational inference with adversarial training [S82,S83]; physics-informed neural networks integrate domain knowledge into generative processes [S83]; and Paul et al. [S84] combined simulation-based inference with quantitative systems pharmacology modeling for virtual RA patients. Propensity score methods with variational autoencoders have been shown to address confounding in observational data [S85-S98].

Table 3. Overview of synthetic data generation methods

**Validation of Synthetic Data**

The value of any synthetic dataset rests on three interdependent properties that have become the de facto evaluation standard: fidelity, utility, and privacy. Fidelity measures how closely synthetic data mirrors real data distributions using metrics such as Jensen-Shannon divergence, Wasserstein distance, maximum mean discrepancy, and correlation matrix comparison [S99,S100-S117]. The Synthetic Data Vault framework has played an important role in standardizing fidelity assessment [S101], though metrics vary substantially across studies.

Utility evaluation assesses whether synthetic data supports its intended analytical purpose. The Train-Synthetic-Test-Real (TSTR) paradigm has emerged as the gold standard, where models trained on synthetic data are tested on held-out real data [S102,S118-S121]. Broader utility measures encompass the replication of epidemiological associations and preservation of clinical effect sizes [S103,S104,S108,S116,S118,S122,S123]. A consistent finding is that while synthetic data closely replicates aggregate statistics, individual-level predictive accuracy may degrade in high-dimensional settings [S113,S124].

Privacy evaluation employs attack-based metrics including membership inference, singling-out risk, attribute disclosure, and nearest-neighbor distance [S124-S151]. Differential privacy provides the strongest formal guarantees but typically degrades utility in proportion to the privacy guarantee's strength [S15,S152]. Most studies employ only one or two privacy metrics and rarely conduct adversarial simulations [S153]. The three dimensions are locked in a fundamental tension: maximizing fidelity increases re-identification risk, while strong privacy degrades both fidelity and utility [S124,S135,S147,S154]. Despite this tension's centrality, only a minority of published studies evaluate all three dimensions simultaneously, with most neglecting privacy entirely.

**Applications in Healthcare**

Five principal application domains have emerged from this diverse methodological foundation. Data augmentation for machine learning training represents the most prevalent use, particularly relevant in clinical domains where labeled data is scarce due to low disease prevalence, high annotation costs, or privacy constraints. Synthetic data augmentation has yielded measurable improvements across a broad range of tasks including low-prevalence disease classification, adverse drug event detection, and clinical outcome prediction [S13,S14,S155,S16-S18,S20-S22,S24-S38,S156-S158]. Applications extend to developmental disorder identification, fall detection algorithms, and wearable-based health monitoring [S159,S160-S165], suggesting that augmentation may represent the most immediately accessible entry point for synthetic data in healthcare, as it requires relatively modest generative quality and faces fewer regulatory hurdles than applications involving data sharing.

A more ambitious application leverages synthetic data to enable multi-institutional research without transferring real patient records, circumventing the privacy and regulatory barriers that have long impeded collaborative clinical research [S15,S124-S141,S143-S147,S149-S152,S166-S171]. In this paradigm, institutions generate synthetic versions of their datasets that preserve analytical properties while eliminating identifiable information, allowing the resulting data to be shared across organizational and national boundaries. Federated analysis systems have begun incorporating synthetic data capabilities [S124,S138,S140], and cross-border sharing within the European Health Data Space represents an active development area [S136,S166].

Digital twins, defined as computational models that replicate individual patients or populations in silico, represent perhaps the most transformative application [S84,S172-S207]. By constructing mechanistic or data-driven models of disease processes and drug responses, digital twin frameworks enable virtual clinical trials that explore treatment strategies without the cost and ethical constraints of traditional recruitment. TWIN-GPT has leveraged LLMs for digital twin generation [S207], and synthetic control arms address single-arm trials where recruitment is limited [S179]. QSP models have already enabled drug development decisions in RA [S84,S175]. For rheumatology, where low-prevalence diseases and heterogeneous populations make large-scale randomized trials exceptionally difficult, digital twin technology holds particular promise.

Medical image synthesis spans virtually every major modality including MRI, CT, X-ray, histopathology, and echocardiography [S13,S21,S22,S24-S29,S31,S32,S35-S38,S156], with cross-modality translation offering radiation dose reduction and cost savings [S155,S32]. Diffusion models have enabled 3D volumetric synthesis [S46]. Synthetic electronic health record generation, combining structured fields, unstructured text, and temporal dynamics, spans the full methodological spectrum from statistical approaches through GANs to LLMs producing longitudinal records with temporal coherence [S14,S60,S61,S103,S106,S118,S119,S121,S208]. Rigorous validation has demonstrated utility for clinical prediction and epidemiological analysis, though performance varies depending on data complexity [S103,S118,S121,S122].

## Supplementary Table S1. Quality Assessment of 13 Rheumatology-Specific Studies

**Quality assessment checklist items:**

- **Q1 (Study design):** Is the study design clearly described and appropriate for the research question?
- **Q2 (Data description):** Is the training data adequately described (source, size, patient characteristics, inclusion/exclusion criteria)?
- **Q3 (Method description):** Is the synthetic data generation method described in sufficient detail to be reproduced?
- **Q4 (Fidelity evaluation):** Is fidelity evaluated using statistical metrics comparing synthetic and real data distributions?
- **Q5 (Utility evaluation):** Is utility demonstrated through downstream task performance or preservation of clinical associations?
- **Q6 (Privacy evaluation):** Is privacy formally evaluated using re-identification risk metrics, membership inference attacks, or disclosure analysis?
- **Q7 (Comparison):** Is the proposed method compared against alternative approaches or established baselines?
- **Q8 (Clinical relevance):** Does the study address a clinically relevant question or demonstrate clinical applicability?
- **Q9 (Limitations):** Does the study discuss its limitations?
- **Q10 (Reproducibility):** Are code, synthetic data, or sufficient implementation details shared to enable reproducibility?

**Scoring:** Y = Yes (fully met), P = Partial (partially met), N = No (not met). Overall quality: High (≥7 Yes), Moderate (4–6 Yes), Low (≤3 Yes).

| Author (Year) | Disease | Q1 | Q2 | Q3 | Q4 | Q5 | Q6 | Q7 | Q8 | Q9 | Q10 | Overall |
| --- | --- | --- | --- | --- | --- | --- | --- | --- | --- | --- | --- | --- |
| Bedathuru (2024) | RA | Y | P | Y | Y | Y | N | P | Y | Y | Y | **High** |
| Zerrouk (2024) | RA | Y | P | P | P | Y | N | N | Y | N | N | Moderate |
| Paul (2024) | RA | Y | P | P | N | P | N | P | Y | N | N | Moderate |
| Prezja (2022) | OA | Y | P | P | P | Y | N | N | Y | N | N | Moderate |
| Jayakumar (2025) | OA | Y | P | N | N | Y | N | Y | Y | N | P | Moderate |
| Coleman (2025) | OA | Y | P | P | P | P | N | N | Y | P | N | Moderate |
| Jiang (2021) | SSc | Y | P | P | Y | Y | Y | Y | Y | N | P | Moderate |
| Jia (2024) | SSc | Y | P | P | N | Y | N | P | Y | P | N | Moderate |
| Sun (2023) | AS | Y | P | P | Y | P | Y | N | P | N | N | Moderate |
| Pezoulas (2023) | SjS | P | N | N | N | P | N | N | P | N | N | Low |
| Pezoulas (2021) | SjS | N | P | Y | Y | N | P | N | N | N | P | Low |
| Xie (2024) | SLE | N | N | N | N | P | N | N | P | N | N | Low |
| Liu (2023) | SLE | N | N | N | N | P | N | N | P | N | N | Low |

**Supplementary Table S2. Overview of Synthetic Data Generation Methods**

| Method | Description | Key Variants | References |
| --- | --- | --- | --- |
| Bayesian Networks | Statistical models encoding conditional dependencies between variables via directed acyclic graphs; generate synthetic records by sampling from learned joint distributions | BN-based synthesis | [S1-S4] |
| Copula Models | Flexible statistical models that capture dependency structures between variables independently from their marginal distributions | Gaussian copula, vine copula | [S5-S10] |
| Gaussian Mixture Models (GMMs) | Probabilistic models representing data as a weighted mixture of Gaussian distributions; capture multimodal populations | BGMM | [S5-S10] |
| Generative Adversarial Networks (GANs) | Two neural networks (generator, discriminator) trained adversarially; the generator creates synthetic samples while the discriminator learns to distinguish real from synthetic | CTGAN, TVAE, ac-GAN, discGAN, DP-GAN | [S209,S210,S13-S38,S155,S211] |
| Diffusion Models | Generative models that learn to reverse a gradual noising process applied to real data, producing new samples through iterative denoising | TabDDPM, CDDPM | [S39-S47] |
| Rule-Based Simulators | Platforms generating synthetic patient trajectories from explicitly coded clinical and epidemiological knowledge rather than learned distributions | Synthea, domain-specific simulators | [S48-S57] |
| Large Language Models (LLMs) | Transformer-based models generating semantically coherent clinical text and structured health records by leveraging contextual reasoning | CEHR-GPT, PromptEHR | [S58-S81] |
| Variational Autoencoders (VAEs) | Neural networks learning a compressed latent representation of data; generate new samples by sampling and decoding from the latent space | VAE-GAN, TVAE | [S82,S83,S95] |
| Physics-Informed Neural Networks (PINNs) | Neural networks with loss functions or architectures constrained to respect known physical, biological, or pharmacological laws | QSP-integrated models | [S83,S84] |
| Digital Twin Frameworks | Mechanistic or data-driven computational models of individual patients; enable virtual population generation through simulation | Boolean network models, multiscale models | [S84,S172-S207] |
| Hybrid Methods | Approaches combining multiple generative paradigms (e.g., VAE+GAN, SBI+QSP, propensity scores+VAE) to leverage complementary strengths | VAE-GAN, SBI+QSP | [S82-S98] |

**Supplementary References**

S1. Gallos P, Matragkas N, Islam SU, et al. INSAFEDARE project: Innovative applications of assessment and assurance of data and synthetic data for regulatory decision support. Stud Health Technol Inform 2024.

S2. Gutsche A, Salameh P, Jahandideh SS, et al. Can synthetic data allow for smaller sample sizes in chronic urticaria research? Clinical & Translational All 2025;15.

S3. Hahn W, Schütte K, Schultz K, et al. Contribution of synthetic data generation towards an improved patient stratification in palliative care. JPM 2022;12:1278.

S4. Pezoulas VC, Grigoriadis GI, Gkois G, et al. A computational pipeline for data augmentation towards the improvement of disease classification and risk stratification models: A case study in two clinical domains. Comput Biol Med 2021;134:104520.

S5. Karimian Sichani E, Smith A, El Emam K, et al. Creating high-quality synthetic health data: Framework for model development and validation. JMIR Form Res 2024;8:e53241.

S6. LeBien J, Velev J, Roche-Lima A. RINet: Synthetic data training for indirect estimation of clinical reference distributions. J Biomed Inform 2026;174:104980.

S7. Rodriguez-Almeida AJ, Fabelo H, Ortega S, et al. Synthetic patient data generation and evaluation in disease prediction using small and imbalanced datasets. IEEE J Biomed Health Inform 2023;27:2670-2680.

S8. van Dijk B, Islam SU, Achterberg J, et al. A novel taxonomy for navigating and classifying synthetic data in healthcare applications. Stud Health Technol Inform 2024.

S9. Vilardell M, Buxó M, Clèries R, et al. Missing data imputation and synthetic data simulation through modeling graphical probabilistic dependencies between variables (modgraprodep): An application to breast cancer survival. Artif Intell Med 2020;107:101875.

S10. Woillard J, Benoist C, Destere A, et al. To be or not to be, when synthetic data meet clinical pharmacology: A focused study on pharmacogenetics. CPT Pharmacom & Syst Pharma 2025;14:82-94.

S11. Kumari C, Siddharthan R. MMM and MMMSynth: Clustering of heterogeneous tabular data, and synthetic data generation. PLoS One 2024;19:e0302271.

S12. Pezoulas VC, Exarchos TP, Tachos NS, et al. Boosting the performance of MALT lymphoma classification in patients with primary sjögren’s syndrome through data augmentation: A case study. Annu Int Conf IEEE Eng Med Biol Soc 2023.

S13. Tagmatova Z, Abdusalomov A, Nasimov R, et al. New approach for generating synthetic medical data to predict type 2 diabetes. Bioengineering 2023;10:1031.

S14. Zhang Y, Dong JL, Xue B, et al. Exploring the utilization of synthetic data in unsupervised clustering for opioid misuse analysis. AMIA Annu Symp Proc 2024;2024:1313-1322.

S15. Torfi A, Fox EA, Reddy CK. Differentially private synthetic medical data generation using convolutional GANs. Inf Sci 2022;586:485-500.

S16. Abidoye I, Ikeji F, Coupland CA, et al. Platelets image classification through data augmentation: A comparative study of traditional imaging augmentation and GAN-Based synthetic data generation techniques using CNNs. J. Imaging 2025;11:183.

S17. Akiya I, Ishihara T, Yamamoto K. Comparison of synthetic data generation techniques for control group survival data in oncology clinical trials: Simulation study. JMIR Med Inform 2024;12:e55118-e55118.

S18. Hu Y, He H, Chen Q, et al. Facilitating clinical information extraction with synthetic data and ontology using large language models. AMIA Annu Symp Proc 2024;2024:500-505.

S19. Assaf JF, Yazbeck H, Reinstein DZ, et al. Enhancing the automated detection of implantable collamer lens vault using generative adversarial networks and synthetic data on optical coherence tomography. J Refract Surg 2024;40.

S20. Coleman LJ, Byrne JL, Edwards S, et al. Patient-specific variability in interleukin-6 and myeloperoxidase responses in osteoarthritis: Insights from synthetic data and clustering analysis. JPM 2025;15:17.

S21. D'Amico S, Dall'Olio D, Sala C, et al. Synthetic data generation by artificial intelligence to accelerate research and precision medicine in hematology. JCO Clin Cancer Inform 2023.

S22. Fuentes D, McSpadden D, Adewole S. Distributed conditional GAN (discgan) for synthetic healthcare data generation. arXiv preprint 2023. arXiv: 2304.04290.

S23. Fok WYR, Fieselmann A, Huemmer C, et al. Adversarial robustness improvement for x-ray bone segmentation using synthetic data created from computed tomography scans. Sci Rep 2024;14.

S24. Kim J, Woo S, Kim T, et al. Development of a cerebellar ataxia diagnosis model using conditional GAN-based synthetic data generation for visuomotor adaptation task. BMC Med Inform Decis Mak 2024;24.

S25. Kuo NI, Garcia F, Sönnerborg A, et al. Generating synthetic clinical data that capture class imbalanced distributions with generative adversarial networks: Example using antiretroviral therapy for HIV. J Biomed Inform 2023;144:104436.

S26. Lacasa M, Prados F, Alegre J, et al. A synthetic data generation system for myalgic encephalomyelitis/chronic fatigue syndrome questionnaires. Sci Rep 2023;13.

S27. Liu R, Wang T, Li H, et al. TMM-Nets: Transferred multi- to mono-modal generation for lupus retinopathy diagnosis. IEEE Trans. Med. Imaging 2023;42:1083-1094.

S28. Mahmoud AY, Neagu D, Scrimieri D, et al. Early diagnosis and personalised treatment focusing on synthetic data modelling: Novel visual learning approach in healthcare. Comput Biol Med 2023;164:107295.

S29. Mahendra M, Umesh C, Bej S, et al. Convex space learning for tabular synthetic data generation. arXiv preprint 2024. arXiv: 2407.09789.

S30. Moya-Sáez E, Peña-Nogales Ó, Luis-García RD, et al. A deep learning approach for synthetic MRI based on two routine sequences and training with synthetic data. Comput Methods Programs Biomed 2021;210:106371.

S31. Mwigereri DG, Kamotho NT, Waljee AK, et al. Synthetic data generation of health and demographic surveillance systems data: A case study in a low- and middle-income country. JAMIA Open 2025;8.

S32. Ashrafi N, Schmitt V, Spang RP, et al. Protect and extend -- using GANs for synthetic data generation of time-series medical records. arXiv preprint 2024. arXiv: 2402.14042.

S33. Parise O, Kronenberger R, Parise G, et al. CTGAN-driven synthetic data generation: A multidisciplinary, expert-guided approach (TIMA). Comput Methods Programs Biomed 2025;259:108523.

S34. Prezja F, Paloneva J, Pölönen I, et al. DeepFake knee osteoarthritis x-rays from generative adversarial neural networks deceive medical experts and offer augmentation potential to automatic classification. Sci Rep 2022;12:18573.

S35. Rafiei A, Rad MG, Sikora A, et al. Improving irregular temporal modeling by integrating synthetic data to the electronic medical record using conditional GANs: A case study of fluid overload prediction in the intensive care unit. 2023.

S36. Scroggins JK, Topaz M, Song J, et al. Does synthetic data augmentation improve the performances of machine learning classifiers for identifying health problems in patient–nurse verbal communications in home healthcare settings? J of Nursing Scholarship 2024;57:47-58.

S37. Kikuchi T, Hanaoka S, Nakao T, et al. Method for generating synthetic data combining chest radiography images with tabular clinical information using dual generative models. arXiv preprint 2023. arXiv: 2308.07573.

S38. Tu L, Choi HHF, Clark H, et al. An approach for cancer outcomes modelling using a comprehensive synthetic dataset. Phys Eng Sci Med 2025;48:1473-1483.

S39. Ebert N, Stricker D, Wasenmüller O. Enhancing robustness and generalization in microbiological few-shot detection through synthetic data generation and contrastive learning. Comput Biol Med 2025;191:110141.

S40. Behal K, Chen J, Fikes C, et al. MCRAGE: Synthetic healthcare data for fairness. arXiv preprint 2023. arXiv: 2310.18430.

S41. Kong H, Kim JY, Moon H, et al. Automation of generative adversarial network-based synthetic data-augmentation for maximizing the diagnostic performance with paranasal imaging. Sci Rep 2022;12.

S42. Moroianu SL, Bluethgen C, Chambon P, et al. Improving performance, robustness, and fairness of radiographic AI models with finely-controllable synthetic data. 2025.

S43. Yoon S, Song S, Jin P, et al. Cascaded 3D diffusion models for whole-body 3D 18-F FDG PET/CT synthesis from demographics. arXiv preprint 2025. arXiv: 2505.22489.

S44. Tian M, Chen B, Guo A, et al. Reliable generation of privacy-preserving synthetic electronic health record time series via diffusion models. J Am Med Inform Assoc 2024;31:2529-2539.

S45. Pozzi M, Noei S, Robbi E, et al. Generating and evaluating synthetic data in digital pathology through diffusion models. Sci Rep 2024;14.

S46. Yan C, Zhang Z, Nyemba S, et al. Generating synthetic electronic health record data using generative adversarial networks: Tutorial. JMIR AI 2024;3:e52615.

S47. Uppalapati K, Abdulkareem S, Yimenicioglu B. RareGraph-Synth: Knowledge-guided diffusion models for generating privacy-preserving synthetic patient trajectories in ultra-rare diseases. arXiv preprint 2025. arXiv: 2510.06267.

S48. Torres-Silva EA, Gaviria-Jiménez JJ, Guevara-Zambrano AM, et al. Datos sintéticos de un modelo de datos común para las aplicaciones de inteligencia artificial en salud materna: Reporte de experiencia en el contexto colombiano. Biomed. 2025;45:71-92.

S49. Alloza C, Knox B, Raad H, et al. A case for synthetic data in regulatory decision‐making in europe. Clin Pharma and Therapeutics 2023;114:795-801.

S50. Cruz-Parada E, Vivar-Estudillo G, Pérez-Campos Mayoral L, et al. Proof-of-concept machine learning framework for arboviral disease classification using literature-derived synthetic data: Methodological development preceding clinical validation. Healthcare 2026;14:247.

S51. Demirel M, Mills B, Gaughan E, et al. Eminet: Moving bacteria detection on optical endomicroscopy images trained on synthetic data. Comput Biol Med 2025;196:110678.

S52. Guruge K, Padmanabha P, Herath HMKKMB, et al. Metaacupoint: Metahuman-generated synthetic data for hand acupoint localization. Healthcare 2025;13:3093.

S53. Haddad T, Kumarapeli P, Khaddaj S. Design and development of SPADE: A synthetic patient data generator for testing healthcare data processing system capabilities. Stud Health Technol Inform 2025.

S54. Lee YG, Kwak M, Kim JE, et al. Synthetic data production for biomedical research. PHRP 2025;16:94-99.

S55. Segal B, Fieggen J, Clifton DA, et al. Bridging the generalisation gap: Synthetic data generation for multi-site clinical model validation. Annu Int Conf IEEE Eng Med Biol Soc 2025.

S56. Walonoski J, Klaus S, Granger E, et al. Synthea™ novel coronavirus (COVID-19) model and synthetic data set. Intelligence-Based Medicine 2020;1-2:100007.

S57. Wiedekopf J, Ulrich H, Essenwanger A, et al. Desiderata for a synthetic clinical data generator. Stud Health Technol Inform 2021.

S58. Smolyak D, Bjarnadóttir MV, Crowley K, et al. Large language models and synthetic health data: Progress and prospects. JAMIA Open 2024;7.

S59. Pang C, Jiang X, Pavinkurve NP, et al. CEHR-GPT: Generating electronic health records with chronological patient timelines. arXiv preprint 2024. arXiv: 2402.04400.

S60. Chen J, Chun D, Patel M, et al. The validity of synthetic clinical data: A validation study of a leading synthetic data generator (Synthea) using clinical quality measures. BMC Med Inform Decis Mak 2019;19.

S61. Nagesh N, Wang Z, Rahmani AM. FairCauseSyn: Towards causally fair LLM-augmented synthetic data generation. Annu Int Conf IEEE Eng Med Biol Soc 2025.

S62. Achuthan S, Chatterjee R, Kotnala S, et al. Leveraging deep learning algorithms for synthetic data generation to design and analyze biological networks. J Biosci 2022;47:43.

S63. Ilaty A, Shirazi H, Homayouni H. SynLLM: A comparative analysis of large language models for medical tabular synthetic data generation via prompt engineering. arXiv preprint 2025. arXiv: 2508.08529.

S64. Brekke PH, Rama T, Pilán I, et al. Synthetic data for annotation and extraction of family history information from clinical text. J Biomed Semant 2021;12.

S65. Draghi B, Wang Z, Myles P, et al. Identifying and handling data bias within primary healthcare data using synthetic data generators. Heliyon 2024;10:e24164.

S66. Freise N, Heitlinger M, Nuredini R, et al. The role of domain-specific models for synthetic data generation with iterative prompt optimization. Annu Int Conf IEEE Eng Med Biol Soc 2025.

S67. Garg M, Liu X, Jeon E, et al. Leveraging reddit data for context-enhanced synthetic health data generation to identify low self esteem. 2025.

S68. Tornqvist M, Zucker J, Fauvel T, et al. A text-to-tabular approach to generate synthetic patient data using LLMs. arXiv preprint 2024. arXiv: 2412.05153.

S69. Pandita A, Keniston A, Madhuripan N. Synthetic data trained open-source language models are feasible alternatives to proprietary models for radiology reporting. NPJ Digit Med 2025;8.

S70. Nalela P. Leveraging generative AI through prompt engineering and rigorous validation to create comprehensive synthetic datasets for AI training in healthcare. arXiv preprint 2025. arXiv: 2504.20921.

S71. Tang R, Han X, Jiang X, et al. Does synthetic data generation of LLMs help clinical text mining? arXiv preprint 2023. arXiv: 2303.04360.

S72. Li R, Wang X, Yu H. DualAlign: Generating clinically grounded synthetic data. arXiv preprint 2025. arXiv: 2509.10538.

S73. Touati H, Thabet R, Fontanili F, et al. AI-driven approach for creating and evaluating a synthetic dataset for medication errors. J Biomed Inform 2025;169:104889.

S74. Das T, Albassam D, Sun J. Synthetic patient-physician dialogue generation from clinical notes using LLM. arXiv preprint 2024. arXiv: 2408.06285.

S75. Xu J, Wang R, Shang S, et al. Chatemg: Synthetic data generation to control a robotic hand orthosis for stroke. IEEE Robot. Autom. Lett. 2025;10:907-914.

S76. Ling Y, Jiang X, Kim Y. MALLM-GAN: Multi-agent large language model as generative adversarial network for synthesizing tabular data. arXiv preprint 2024. arXiv: 2406.10521.

S77. Lin Y, Yu ZB, Lee S. A case study exploring the current landscape of synthetic medical record generation with commercial LLMs. arXiv preprint 2025. arXiv: 2504.14657.

S78. Zheng H, Ai X, Liu X, et al. Profile-aided distillation framework for personalized sleep analysis with compact models using LLM-guided synthetic data. Front Physiol 2026;16.

S79. Wang Z, Sun J. PromptEHR: Conditional electronic healthcare records generation with prompt learning. arXiv preprint 2022. arXiv: 2211.01761.

S80. Zolnour A, Azadmaleki H, Haghbin Y, et al. LLMCARE: Early detection of cognitive impairment via transformer models enhanced by LLM-generated synthetic data. Front Artif Intell 2025;8.

S81. Lorge I, Joyce DW, Taylor N, et al. Detecting the clinical features of difficult-to-treat depression using synthetic data from large language models. Comput Biol Med 2025;194:110246.

S82. Farhadyar K, Bonofiglio F, Hackenberg M, et al. Combining propensity score methods with variational autoencoders for generating synthetic data in presence of latent sub-groups. BMC Med Res Methodol 2024;24.

S83. Apellániz PA, Parras J, Zazo S. An improved tabular data generator with VAE-GMM integration. arXiv preprint 2024. arXiv: 2404.08434.

S84. Paul N, Karamitsou V, Giegerich C, et al. Building virtual patients using simulation-based inference. Front. Syst. Biol. 2024;4.

S85. Dedhia B, Balasubramanian R, Jha NK. Scout: Synthetic counterfactuals via spatiotemporal transformers for actionable healthcare. arXiv preprint 2022. arXiv: 2207.04208.

S86. Umesh C, Schultz K, Mahendra M, et al. Dependency-aware synthetic tabular data generation. arXiv preprint 2025. arXiv: 2507.19211.

S87. Gao C, Killeen BD, Hu Y, et al. Synthetic data accelerates the development of generalizable learning-based algorithms for x-ray image analysis. Nat Mach Intell 2023;5:294-308.

S88. Grössler M, Ückert F, Riemann LT. Open-source synthetic data generation of clinical routine data. Stud Health Technol Inform 2025.

S89. Zhou G, Barbieri S. Generating clinically realistic EHR data via a hierarchy- and semantics-guided transformer. arXiv preprint 2025. arXiv: 2502.20719.

S90. Hao J, He X, Durak G, et al. Coronary artery segmentation in non-contrast cardiac CT using anatomy-informed contrastive learning and synthetic data. Phys. Med. Biol. 2026;71:025017.

S91. Hwang EE, Rivera ML, Jia L, et al. Deep learning detection of retinitis pigmentosa inheritance forms through synthetic data expansion of a rare disease dataset. 2025.

S92. Oono K, Charoenphakdee N, Bito K, et al. Virtual human generative model: Masked modeling approach for learning human characteristics. arXiv preprint 2023. arXiv: 2306.10656.

S93. Petrakos NZ, Moodie EEM, Savy N. A framework for generating realistic synthetic tabular data in a randomized controlled trial setting. arXiv preprint 2025. arXiv: 2501.17719.

S94. Schulz NA, Carus J, Wiederhold AJ, et al. Learning debiased graph representations from the OMOP common data model for synthetic data generation. BMC Med Res Methodol 2024;24.

S95. Xie Q, Li N, Lu Y, et al. Prediction of treatment effect of SLE-ITP patients based on cost-sensitive neural network and variational autoencoder. J Clin Rheumatol 2024;30:e78-e85.

S96. Yang H, Olefir I, Tzoumas S, et al. Synthetic data framework to estimate the minimum detectable concentration of contrast agents for multispectral optoacoustic imaging of small animals. Journal of Biophotonics 2019;12.

S97. Zazzetti E, D'Amico S, Jacobs F, et al. Longitudinal synthetic data generation by artificial intelligence to accelerate clinical and translational research in breast cancer. JCO Clin Cancer Inform 2025.

S98. Zhang J, Bousse A, Pham C, et al. Semi-supervised learning for dose prediction in targeted radionuclide therapy: A synthetic data study. Phys. Med. Biol. 2026;71:025005.

S99. Zamzmi G, Subbaswamy A, Sizikova E, et al. Scorecard for synthetic medical data evaluation. Commun Eng 2025;4.

S100. Basri MA, Chen H. How useful is synthetic data in developing predictive models for health? Stud Health Technol Inform 2025.

S101. Belgodere B, Dognin P, Ivankay A, et al. Auditing and generating synthetic data with controllable trust trade-offs. arXiv preprint 2023. arXiv: 2304.10819.

S102. Davalan W, Khalaf R, Diaz RJ. Reproduction of original glioblastoma and brain metastasis research findings using synthetic data. World Neurosurgery 2025;196:123808.

S103. Foppa C, D’Amico S, Delleani M, et al. Synthetic data generated by artificial intelligence to optimize surgical trial design. Ann Surg 2025;282:810-817.

S104. Visani G, Graffi G, Alfero M, et al. Enabling synthetic data adoption in regulated domains. arXiv preprint 2022. arXiv: 2204.06297.

S105. Goncalves A, Ray P, Soper B, et al. Generation and evaluation of synthetic patient data. BMC Med Res Methodol 2020;20.

S106. Ji E, Ohn JH, Jo H, et al. Evaluating the utility of data integration with synthetic data and statistical matching. Sci Rep 2025;15.

S107. Koul A, Duran D, Hernandez-Boussard T. Synthetic data, synthetic trust: Navigating data challenges in the digital revolution. Lancet Digit Health 2025;7:100924.

S108. Miletic M, Bollinger A, Allemann SS, et al. Synthetic data for pharmacogenetics: Enabling scalable and secure research. JAMIA Open 2025;8.

S109. Mohri O, Seki T, Kawazoe Y, et al. Evaluation of synthetic data generation methods for medical tabular data: Representation of distribution tails. Stud Health Technol Inform 2025.

S110. Nafis N, Esnaola I, Martinez-Perez A, et al. Critical challenges and guidelines in evaluating synthetic tabular data: A systematic review. arXiv preprint 2025. arXiv: 2504.18544.

S111. Pilgram L, El Kababji S, Liu D, et al. Magnitude and impact of hallucinations in tabular synthetic health data on prognostic machine learning models: Validation study. J Med Internet Res 2025;27:e77893.

S112. Prasanna A, Jing B, Plopper G, et al. Synthetic health data can augment community research efforts to better inform the public during emerging pandemics. 2023.

S113. Santangelo G, Nicora G, Bellazzi R, et al. How good is your synthetic data? synthro, a dashboard to evaluate and benchmark synthetic tabular data. BMC Med Inform Decis Mak 2025;25.

S114. Schaudt D, Späte C, von Schwerin R, et al. A critical assessment of generative models for synthetic data augmentation on limited pneumonia x-ray data. Bioengineering 2023;10:1421.

S115. BN S, Mattioli D, Abdullah S, et al. How real are synthetic therapy conversations? evaluating fidelity in prolonged exposure dialogues. arXiv preprint 2025. arXiv: 2504.21800.

S116. Suresh K, Cohen MS, Hartnick CJ, et al. Generation of synthetic tympanic membrane images: Development, human validation, and clinical implications of synthetic data. PLOS Digit Health 2023;2:e0000202.

S117. Vallevik VB, Babic A, Marshall SE, et al. Can i trust my fake data - a comprehensive quality assessment framework for synthetic tabular data in healthcare. Int J Med Inform 2024;185:105413.

S118. El Emam K, Mosquera L, Fang X. Validating a membership disclosure metric for synthetic health data. JAMIA Open 2022;5.

S119. Yu K, Ishikura S, Usukura Y, et al. SHAP distance: An explainability-aware metric for evaluating the semantic fidelity of synthetic tabular data. arXiv preprint 2025. arXiv: 2511.17590.

S120. Miletic M, Sariyar M. Utility-based analysis of statistical approaches and deep learning models for synthetic data generation with focus on correlation structures: Algorithm development and validation. JMIR AI 2025;4:e65729.

S121. Teeple S, Muñoz LE, Aysola J. Synthetic data and health equity: Accounting for racism and sexism in health care delivery. Health Aff Sch 2025;3.

S122. El Emam K, Mosquera L, Fang X, et al. Utility metrics for evaluating synthetic health data generation methods: Validation study. JMIR Med Inform 2022;10:e35734.

S123. Kühnel L, Schneider J, Perrar I, et al. Synthetic data generation for a longitudinal cohort study – evaluation, method extension and reproduction of published data analysis results. Sci Rep 2024;14.

S124. Adams T, Birkenbihl C, Otte K, et al. On the fidelity versus privacy and utility trade-off of synthetic patient data. iScience 2025;28:112382.

S125. Arora A, Wagner SK, Carpenter R, et al. The urgent need to accelerate synthetic data privacy frameworks for medical research. Lancet Digit Health 2025;7:e157-e160.

S126. Chhoa H, Chabriat H, Anato AJ, et al. Improvement of an external predictive model based on new information using a synthetic data approach. Neurol Genet 2023;9.

S127. El Emam K, Mosquera L, Bass J. Evaluating identity disclosure risk in fully synthetic health data: Model development and validation. J Med Internet Res 2020;22:e23139.

S128. German E, Samira D, Elovici Y, et al. MIA-EPT: Membership inference attack via error prediction for tabular data. arXiv preprint 2025. arXiv: 2509.13046.

S129. Guépin F, Meeus M, Cretu A, et al. Synthetic is all you need: Removing the auxiliary data assumption for membership inference attacks against synthetic data. arXiv preprint 2023. arXiv: 2307.01701.

S130. Giouroukou K, Marias K, Tsiknakis M, et al. Rethinking privacy in medical imaging AI: From metadata and pixel-level identification risks to federated learning and synthetic data challenges. Radiol Artif Intell 2026;8.

S131. Guillaudeux M, Rousseau O, Petot J, et al. Patient-centric synthetic data generation, no reason to risk re-identification in biomedical data analysis. NPJ Digit Med 2023;6.

S132. Guo A, Foraker RE, MacGregor RM, et al. The use of synthetic electronic health record data and deep learning to improve timing of high-risk heart failure surgical intervention by predicting proximity to catastrophic decompensation. Front Digit Health 2020;2.

S133. Jeanson F, Farkouh ME, Godoy LC, et al. Medical calculators derived synthetic cohorts: A novel method for generating synthetic patient data. Sci Rep 2024;14.

S134. Jiang B, Raftery AE, Steele RJ, et al. Balancing inferential integrity and disclosure risk via model targeted masking and multiple imputation. Journal of the American Statistical Association 2021;117:52-66.

S135. Juwara L, El-Hussuna A, El Emam K. An evaluation of synthetic data augmentation for mitigating covariate bias in health data. Patterns 2024;5:100946.

S136. Kaabachi B, Despraz J, Meurers T, et al. A scoping review of privacy and utility metrics in medical synthetic data. NPJ Digit Med 2025;8.

S137. Kafatos G, Levy J, Jose S, et al. Leveraging synthetic data to facilitate research: A collaborative model for analyzing sensitive national cancer registry data in england. Ther Innov Regul Sci 2025;59:919-928.

S138. Lun R, Siegal D, Ramsay T, et al. Synthetic data in cancer and cerebrovascular disease research: A novel approach to big data. PLoS One 2024;19:e0295921.

S139. Krishnamoorthy MV. Data obfuscation through latent space projection (LSP) for privacy-preserving AI governance: Case studies in medical diagnosis and finance fraud detection. arXiv preprint 2024. arXiv: 2410.17459.

S140. Dombrowski M, Reynaud H, Kainz B. Uncovering hidden subspaces in video diffusion models using re-identification. arXiv preprint 2024. arXiv: 2411.04956.

S141. Moazemi S, Adams T, Ng HG, et al. Nfdi4health workflow and service for synthetic data generation, assessment and risk management. Stud Health Technol Inform 2024.

S142. Mendelevitch O, Lesh MD. Fidelity and privacy of synthetic medical data. arXiv preprint 2021. arXiv: 2101.08658.

S143. Pilgram L, Ko H, Tung A, et al. Protecting patient privacy in tabular synthetic health data: A regulatory perspective. NPJ Digit Med 2025;8.

S144. Prediger L, Jälkö J, Honkela A, et al. Collaborative learning from distributed data with differentially private synthetic data. BMC Med Inform Decis Mak 2024;24.

S145. Reiner Benaim A, Almog R, Gorelik Y, et al. Analyzing medical research results based on synthetic data and their relation to real data results: Systematic comparison from five observational studies. JMIR Med Inform 2020;8:e16492.

S146. Resnick DM, Cox CS, Mirel LB. Using synthetic data to replace linkage derived elements: A case study. Health Serv Outcomes Res Method 2021;21:389-406.

S147. Mustaqim SM, Kotal A, Yi PH. When privacy isn't synthetic: Hidden data leakage in generative AI models. arXiv preprint 2025. arXiv: 2512.06062.

S148. Shi J, Wang D, Tesei G, et al. Generating high-fidelity privacy-conscious synthetic patient data for causal effect estimation with multiple treatments. Front Artif Intell 2022;5.

S149. Hyrup T, Lautrup AD, Zimek A, et al. Sharing is cairing: Characterizing principles and assessing properties of universal privacy evaluation for synthetic tabular data. Mach Learn Appl 2024;18:100608.

S150. Wang E, Mott K, Zhang H, et al. Validation assessment of privacy‐preserving synthetic electronic health record data: Comparison of original versus synthetic data on real‐world covid‐19 vaccine effectiveness. Pharmacoepidemiology and Drug 2024;33.

S151. Wang EH, Myles P, Foraker R, et al. Crossing borders securely: Synthetic data and federated networks for privacy-preserving access to real-world data and emerging use cases. NPJ Digit Med 2025;8.

S152. Kotal A, Joshi A. Differentially private synthetic data generation using context-aware GANs. Proc IEEE Int Conf Big Data 2024:6289-6297.

S153. Jordon J, Wilson A, Schaar MVD. Synthetic data: Opening the data floodgates to enable faster, more directed development of machine learning methods. arXiv preprint 2020. arXiv: 2012.04580.

S154. Yan C, Yan Y, Wan Z, et al. A multifaceted benchmarking of synthetic electronic health record generation models. Nat Commun 2022;13.

S155. Sun H, Plawinski J, Subramaniam S, et al. A deep learning approach to private data sharing of medical images using conditional generative adversarial networks (GANs). PLoS One 2023;18:e0280316.

S156. Schonfeld E, Veeravagu A. Generative adversarial network based synthetic learning and a novel domain relevant loss term for spine radiographs. arXiv preprint 2022. arXiv: 2205.02843.

S157. Greenberg JK, Landman JM, Kelly MP, et al. Leveraging artificial intelligence and synthetic data derivatives for spine surgery research. Global Spine Journal 2022;13:2409-2421.

S158. Bhardwaj P, Jones C, Dierich L, et al. TabularQGAN: A quantum generative model for tabular data. arXiv preprint 2025. arXiv: 2505.22533.

S159. Rujas M, Martín Gómez del Moral Herranz R, Fico G, et al. Synthetic data generation in healthcare: A scoping review of reviews on domains, motivations, and future applications. Int J Med Inform 2025;195:105763.

S160. Moell B, Aronsson FS. Voice cloning for dysarthric speech synthesis: Addressing data scarcity in speech-language pathology. arXiv preprint 2025. arXiv: 2503.01266.

S161. Debnath M, Alamgeer S, Kabir MS, et al. Enhancing wearable fall detection system via synthetic data. Sensors 2025;25:4639.

S162. Hahn W, Ahmadi N, Hoffmann K, et al. Synthetic data generation in hematology – paving the way for OMOP and FHIR integration. Stud Health Technol Inform 2024.

S163. Jia J, Hernández-Girón I, Schouffoer AA, et al. Explainable fully automated CT scoring of interstitial lung disease for patients suspected of systemic sclerosis by cascaded regression neural networks and its comparison with experts. Sci Rep 2024;14.

S164. Rajotte J, Bergen R, Buckeridge DL, et al. Synthetic data as an enabler for machine learning applications in medicine. iScience 2022;25:105331.

S165. Williams MC, MacArthur JAL, Forsyth R, et al. New ways to use imaging data in cardiovascular research: Survey of opinions on federated learning and synthetic data. Eur Heart J Imaging Methods Pract 2025;3.

S166. Achterberg J, van Dijk B, Islam SU, et al. The data sharing paradox of synthetic data in healthcare. Stud Health Technol Inform 2025.

S167. Kotal A, Joshi A. KIPPS: Knowledge infusion in privacy preserving synthetic data generation. arXiv preprint 2024. arXiv: 2409.17315.

S168. Pezoulas VC, Mylona E, Tachos NS, et al. Utilizing synthetic data for privacy-preserving AI modeling in radiomics: A case study. Annu Int Conf IEEE Eng Med Biol Soc 2025;2025:1-4.

S169. Azizi Z, Lindner S, Shiba Y, et al. A comparison of synthetic data generation and federated analysis for enabling international evaluations of cardiovascular health. Sci Rep 2023;13.

S170. Burgess MA, Hosking B, Reguant R, et al. Privacy-hardened and hallucination-resistant synthetic data generation with logic-solvers. Bioinformatics 2025;41.

S171. Qian Z, Callender T, Cebere B, et al. Synthetic data for privacy-preserving clinical risk prediction. Sci Rep 2024;14.

S172. Apellániz PA, Galende BA, Jiménez A, et al. Advancing cancer research with synthetic data generation in low-data scenarios. IEEE J Biomed Health Inform 2026;30:1666-1679.

S173. Azizi Z, Zheng C, Mosquera L, et al. Can synthetic data be a proxy for real clinical trial data? a validation study. BMJ Open 2021;11:e043497.

S174. Bedathuru D, Rengaswamy M, Channavazzala M, et al. Multiscale, mechanistic model of rheumatoid arthritis to enable decision making in late stage drug development. npj Syst Biol Appl 2024;10.

S175. Cheng Y, Straube R, Alnaif A, et al. Virtual populations for quantitative systems pharmacology models. Methods Mol Biol.

S176. Cogan NG, Bao F, Paus R, et al. Data assimilation of synthetic data as a novel strategy for predicting disease progression in alopecia areata. Math Med Biol 2021;38:314-332.

S177. Bertolini D, Loukianov AD, Smith AM, et al. Modeling disease progression in mild cognitive impairment and Alzheimer's disease with digital twins. arXiv preprint 2020. arXiv: 2012.13455.

S178. Danilov VV, Kolpashchikov DY, Gerget OM, et al. Use of semi-synthetic data for catheter segmentation improvement. Computerized Medical Imaging and Graphics 2023;106:102188.

S179. Dinh T, Dalianis H. Evaluating privacy and utility in synthetic EHR data generation for adverse drug event detection. Stud Health Technol Inform 2025.

S180. Elvatun S, Knoors D, Brant S, et al. Synthetic data as external control arms in scarce single-arm clinical trials. PLOS Digit Health 2025;4:e0000581.

S181. Garbey M, Lesport Q, Kaminski HJ. Bridging the heterogeneity of myasthenia gravis severity scores for digital twin development. 2025.

S182. Garbey M, Lesport Q, Kaminski HJ. Bridging the heterogeneity of myasthenia gravis severity scores for digital twin development. 2025.

S183. Georgiou GP. Enhancing developmental language disorder identification with artificial intelligence: Development of an explainable screening app using real and synthetic data. J Autism Dev Disord 2025.

S184. Chenel H, Marku M, James T, et al. Multilayer network approaches to omics data integration in digital twins for cancer research. arXiv preprint 2024. arXiv: 2410.07252.

S185. Iyer AA, Umadevi KS. Design and analysis of twincardio framework to detect and monitor cardiovascular diseases using digital twin and deep neural network. Sci Rep 2025;15.

S186. Jayakumar P, Rathouz PJ, Lin E, et al. Shared decision making using digital twins in knee osteoarthritis care: A randomized clinical trial of an AI-enabled decision aid versus education alone on decision quality, physical function, and user experience. eClinicalMedicine 2025;89:103545.

S187. Jiang Y, García-Durán A, Losada IB, et al. Generative models for synthetic data generation: Application to pharmacokinetic/pharmacodynamic data. J Pharmacokinet Pharmacodyn 2024;51:877-885.

S188. Johnson LG, Wilson DR, Mulpuri K. A biomechanical digital twin of legg–calvé–perthes disease deformity. Int J CARS 2025.

S189. Walsh JR, Smith AM, Pouliot Y, et al. Generating digital twins with multiple sclerosis using probabilistic neural networks. arXiv preprint 2020. arXiv: 2002.02779.

S190. Camps J, Berg LA, Wang ZJ, et al. Digital twinning of the human ventricular activation sequence to clinical 12-lead ECGs and magnetic resonance imaging using realistic purkinje networks for in silico clinical trials. arXiv preprint 2023. arXiv: 2306.13740.

S191. Camps J, Wang ZJ, Doste R, et al. Cardiac digital twin pipeline for virtual therapy evaluation. arXiv preprint 2024. arXiv: 2401.10029.

S192. Kuang K, Dean F, Jedlicki JB, et al. Med-real2sim: Non-invasive medical digital twins using physics-informed self-supervised learning. arXiv preprint 2024. arXiv: 2403.00177.

S193. Lai TC, Ngorsuraches S. Roles of artificial intelligence--based synthetic data in health economics and outcomes research. Value in Health 2025;28:1690-1695.

S194. Cossio M. A perspective on the use of health digital twins in computational pathology. arXiv preprint 2022. arXiv: 2212.00573.

S195. Marino S, Cassidy R, Nanni J, et al. Medical data sharing and synthetic clinical data generation – maximizing biomedical resource utilization and minimizing participant re-identification risks. NPJ Digit Med 2025;8.

S196. Alam N, Basilico J, Bertolini D, et al. Digital twin generators for disease modeling. arXiv preprint 2024. arXiv: 2405.01488.

S197. Padoan A, Plebani M. Dynamic mirroring: Unveiling the role of digital twins, artificial intelligence and synthetic data for personalized medicine in laboratory medicine. Clin Chem Lab Med 2024;62:2156-2161.

S198. Pezoulas VC, Mylona E, Tachos NS, et al. Utilizing synthetic data for privacy-preserving AI modeling in radiomics: A case study <sup>*</sup>. Annu Int Conf IEEE Eng Med Biol Soc 2025.

S199. Pstras L, Waniewski J. A synthetic dataset for in silico studies of parameters influencing changes in mean arterial blood pressure during hemodialysis *. Annu Int Conf IEEE Eng Med Biol Soc 2025:1-6.

S200. Saffaran S, Kwok TC, Sharkey D, et al. Digital twins of mechanically ventilated preterm neonates with respiratory distress syndrome. arXiv preprint 2025. arXiv: 2509.18999.

S201. Jaffery OA, Lopez-Barrera CE, Rodero C, et al. Automated generation of ablation lesion masks: A unison of electro and optic flow mapping for persistent AF virtual cohorts. Europace 2026;28.

S202. Faruqui SHA, Alaeddini A, Du Y, et al. Nurse-in-the-loop artificial intelligence for precision management of type 2 diabetes in a clinical trial utilizing transfer-learned predictive digital twin. arXiv preprint 2024. arXiv: 2401.02661.

S203. Guarrasi V, Feola FD, Restivo R, et al. Whole-body image-to-image translation for a virtual scanner in a healthcare digital twin. arXiv preprint 2025. arXiv: 2503.15555.

S204. Xing X, Ser JD, Wu Y, et al. HDL: Hybrid deep learning for the synthesis of myocardial velocity maps in digital twins for cardiac analysis. arXiv preprint 2022. arXiv: 2203.05564.

S205. Wang Y, Fu T, Xu Y, et al. TWIN-GPT: Digital twins for clinical trials via large language model. arXiv preprint 2024. arXiv: 2404.01273.

S206. Zerrouk N, Augé F, Niarakis A. Building a modular and multi-cellular virtual twin of the synovial joint in rheumatoid arthritis. NPJ Digit Med 2024;7.

S207. Zhou N, Wang L, Marino S, et al. DataSifter II: Partially synthetic data sharing of sensitive information containing time-varying correlated observations. Journal of Algorithms & Computational Technology 2022;16.

S208. Chen X, Wu Z, Shi X, et al. Generating synthetic electronic health record data: A methodological scoping review with benchmarking on phenotype data and open-source software. J Am Med Inform Assoc 2025;32:1227-1240.

S209. Finetti R, Roncaglia B, Visibelli A, et al. Data augmentation and synthetic data generation in rare disease research: A scoping review. Med Sci (Basel) 2025;13:260.

S210. Pezoulas VC, Tachos NS, Gkois G, et al. Bayesian inference-based gaussian mixture models with optimal components estimation towards large-scale synthetic data generation for in silico clinical trials. IEEE Open J Eng Med Biol 2022;3:108-114.

S211. Ramachandranpillai R, Sikder MF, Bergmeir C, et al. Bt-GAN: Generating fair synthetic healthdata via bias-transforming generative adversarial networks. J Artif Intell Res 2024;80:1015-1054.
